# Macrophage sensing of single-walled carbon nanotubes *via* Toll-like receptors

**DOI:** 10.1038/s41598-018-19521-9

**Published:** 2018-01-18

**Authors:** Sourav P. Mukherjee, Olesja Bondarenko, Pekka Kohonen, Fernando T. Andón, Táňa Brzicová, Isabel Gessner, Sanjay Mathur, Massimo Bottini, Paolo Calligari, Lorenzo Stella, Elena Kisin, Anna Shvedova, Reija Autio, Heli Salminen-Mankonen, Riitta Lahesmaa, Bengt Fadeel

**Affiliations:** 1 Nanosafety & Nanomedicine Laboratory, Division of Molecular Toxicology, Institute of Environmental Medicine, Karolinska Institutet, 17177 Stockholm, Sweden; 2 Laboratory of Environmental Toxicology, National Institute of Chemical Physics and Biophysics, Tallinn, 12618 Estonia; 3 Laboratory of Cellular Immunology, Humanitas Clinical and Research Institute, 20089 Rozzano-Milano, Italy; 4 Department of Genetic Toxicology and Nanotoxicology, Institute of Experimental Medicine AS CR, 14220 Prague, Czech Republic; 5 Inorganic and Materials Chemistry, University of Cologne, 50939 Cologne, Germany; 6 Department of Experimental Medicine and Surgery, University of Rome Tor Vergata, Rome, 00173 Italy; 7 Sanford Burnham Prebys Medical Discovery Institute, La Jolla, CA 92037 USA; 8 Department of Chemical Sciences and Technologies, University of Rome Tor Vergata, Rome, 00133 Italy; 9 Exposure Assessment Branch, National Institute for Occupational Safety and Health, Morgantown, WV 26505 USA; 10 Department Pharmacology & Physiology, West Virginia University, Morgantown, WV 26505 USA; 11 Faculty of Social Sciences, University of Tampere, 33014 Tampere, Finland; 12 Turku Centre for Biotechnology, University of Turku, 20520 Turku, and Åbo Akademi University, 20500 Turku, Finland

## Abstract

Carbon-based nanomaterials including carbon nanotubes (CNTs) have been shown to trigger inflammation. However, how these materials are ‘sensed’ by immune cells is not known. Here we compared the effects of two carbon-based nanomaterials, single-walled CNTs (SWCNTs) and graphene oxide (GO), on primary human monocyte-derived macrophages. Genome-wide transcriptomics assessment was performed at sub-cytotoxic doses. Pathway analysis of the microarray data revealed pronounced effects on chemokine-encoding genes in macrophages exposed to SWCNTs, but not in response to GO, and these results were validated by multiplex array-based cytokine and chemokine profiling. Conditioned medium from SWCNT-exposed cells acted as a chemoattractant for dendritic cells. Chemokine secretion was reduced upon inhibition of NF-κB, as predicted by upstream regulator analysis of the transcriptomics data, and Toll-like receptors (TLRs) and their adaptor molecule, MyD88 were shown to be important for CCL5 secretion. Moreover, a specific role for TLR2/4 was confirmed by using reporter cell lines. Computational studies to elucidate how SWCNTs may interact with TLR4 in the absence of a protein corona suggested that binding is guided mainly by hydrophobic interactions. Taken together, these results imply that CNTs may be ‘sensed’ as pathogens by immune cells.

## Introduction

Carbon-based nanomaterials including carbon nanotubes (CNTs) and graphene oxide (GO) are potential candidates for various applications in medicine such as drug delivery and imaging^1^. However, the successful translation of nanomaterials for biomedical applications requires a detailed understanding of the biological interactions of the materials. In particular, interactions of nanomaterials with the immune system, the first line of defense against foreign intrusion, are of key importance^2^. The innate immune system is deployed in defense against microorganisms and involves the recognition of pathogen-associated molecular patterns (PAMPs) by (PRRs) on the surface of phagocytic cells. The immune system also responds to tissue damage, a process that is triggered by so-called danger or damage-associated molecular patterns (DAMPs)^2^. We previously hypothesized that nanoparticles might be recognized directly as nanoparticle-associated molecular patterns or NAMPs by cells of the immune system^3^. However, experimental evidence to support this idea was largely lacking. Hence, while there is an emerging body of literature on nanomaterial effects on the immune system, there are few if any studies in which evidence for specific ‘sensing’ of nanoparticles by immune-competent cells has been provided. Previous work has shown that proteins adsorbed onto the surface of nanoparticles can activate macrophages *via* surface receptors, resulting in the secretion of pro-inflammatory cytokines^4^, but whether nanomaterials themselves are recognized by immune cells through specific receptors is not known.

Single-walled and multi-walled CNTs as well as carbon nanofibers have been shown to induce pro-inflammatory and pro-fibrotic responses, especially following pulmonary exposure^5^. Specifically, exposure to SWCNTs was shown to elicit acute inflammation and early-onset fibrosis in the lungs of mice, with neutrophil accumulation, followed by macrophage influx, and an early elevation of pro-inflammatory cytokines followed by production of pro-fibrotic cytokines^6^. Moreover, elevated numbers of dendritic cells (DCs) are found in the lungs of mice following pharyngeal aspiration of SWCNTs^7^. Furthermore, based on the literature available at the time, certain rigid and ‘needle-like’ MWCNTs were classified by the International Agency for Research on Cancer (IARC) as being potentially carcinogenic to humans^8^, and a more recent, in-depth examination of *in vivo* and *in vitro* studies has affirmed the original evaluation that some MWCNTs are potentially carcinogenic, while the data are inconclusive for others^9^. For graphene-based materials, a consensus on toxicity or health risks has yet to emerge, although considerable efforts are being invested in order to address this question in a systematic fashion^10,11^. In a recent study, so-called graphene nanoplatelets were shown to induce pulmonary toxicity in mice at high doses, but no lung fibrosis^12^. In another recent inhalation study in rats, graphene nanoplatelets showed low toxicity, with no distinct pathology or inflammation; the materials were ingested by lung macrophages^13^. In a study on single-layer GO sheets with lateral dimensions below 500 nm, no significant cytotoxic responses were noted using A549 lung carcinoma cells, and no inflammation or granuloma formation was observed *in vivo* following intraperitoneal injection^14^, while a more recent study suggested that the impact of GO on human peripheral blood-derived cells was dependent on the lateral dimensions of GO^15^.

Here we present detailed mechanistic *in vitro* studies to address the impact of well-characterized and endotoxin-free SWCNTs and GO on primary human macrophages. Guided by global transcriptomics analysis of macrophages exposed to these materials, we focused our studies on chemokine signaling and could show that SWCNTs, but not GO, induced chemokine secretion in macrophages. Our experimental results suggested that SWCNTs were sensed by Toll-like receptors (TLRs), PRRs on the surface of phagocytic cells^16^. Cellular uptake of SWCNTs was not required for chemokine signaling in exposed macrophages. Molecular docking suggested that SWCNTs may interact with TLR4 both *via* the tip and the side-walls. These studies indicate that immune cells are able to ‘sense’ SWCNTs through specific immune receptors and as such are relevant for our understanding of the impact of these materials on human health.

## Results

### Characterization of carbon-based nanomaterials

SWCNTs, produced by the high pressure CO disproportionation process (HiPco) technique, and GO, synthesized by a modified Hummer’s method, were characterized using an array of analytical techniques. Transmission electron microscopy (TEM) revealed that SWCNTs had an average diameter of 1–4 nm and an average length of 0.5–2 µm, whereas GO had an average diameter (lateral size) of 1.1 ± 0.3 µm. The surface charge (ζ-potential) was −42.3 ± 0.9 mV and −42.0 ± 1.2 mV for SWCNTs and GO, respectively. The samples were also characterized following dispersion in cell culture medium (i.e., DMEM) with and without 10% FBS (Fig. S3a–e). The ζ-potential remained negative in DMEM + FBS, though less negatively charged when compared to samples dispersed in water. DLS measurements suggested that SWCNT and GO were less agglomerated in DMEM + FBS than in medium without FBS, though DLS results for non-spherical objects should be interpreted with caution.

Nanomaterials are frequently contaminated with lipopolysaccharide (LPS) or endotoxin, the cell wall component of Gram-negative bacteria^17^. Therefore, both materials were tested by using the conventional limulus amoebocyte lysate (LAL) assay and found to be endotoxin-free (data not shown). Moreover, in order to exclude potential artefacts due to interference with the assay, which could skew the interpretation of the biological data, we also performed a macrophage activation test based on the evaluation of TNF-α secretion by primary human monocyte-derived macrophages (HMDM) in the presence and absence of a specific LPS inhibitor^18^. LPS (100 ng/mL) was included as a positive control. Using this approach, the CNTs were confirmed to be endotoxin-free, as macrophage secretion of TNF-α in response to CNTs was very low and not affected by polymyxin B (Fig. S1a–c).

### Cytotoxicity assessment and cellular uptake

We then assessed both nanomaterials for cytotoxicity using the lactate dehydrogenase (LDH) release assay. ZnO nanoparticles (100 µg/mL) which are known to be cytotoxic for macrophages^19^, were included as a positive control. SWCNT exposure resulted in a slight, dose-dependent increase in LDH release at 24 h (14–16% more LDH release compared to the control at concentrations 30 and 100 µg/mL, respectively; p < 0.05), whereas GO did not yield any cytotoxicity in HMDMs (Fig. S1d). Next, we assessed for cellular uptake of the nanomaterials. TEM imaging confirmed that both nanomaterials were internalized by HMDM at 24 h (Fig. S2). SWCNTs and GO were mostly found within membrane enclosed vacuoles, suggesting that the uptake had occurred through an active, most likely endocytic process. Engulfment of GO changed its appearance from a 2D structure to a densely packed structure (Fig. S2).

### Microarray analysis of macrophage responses

**Figure 1:**
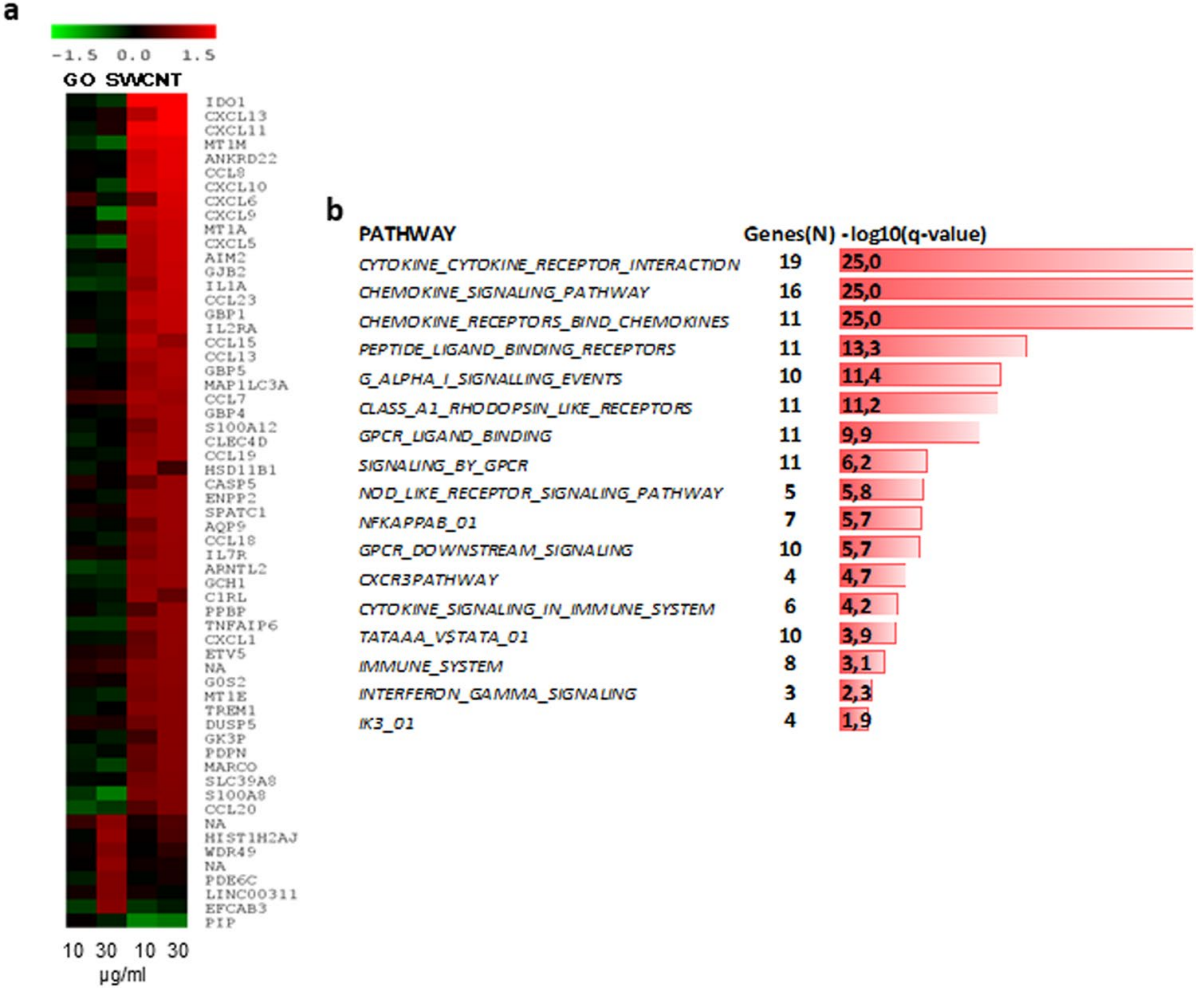
Transcriptomics analysis of macrophages exposed to carbon-based nanomaterials. Global gene expression profiling of human monocyte-derived macrophages (HMDM) was conducted after 24 h exposure to single-walled carbon nanotubes (SWCNTs) or graphene oxide (GO) (see Supplementary Table 1). (**a**) Heatmap of differently expressed genes in response to 10 and 30 µg/mL GO (line 1 and 2, respectively) or 10 and 30 µg/mL SWCNTs (line 3 and 4, respectively). Upregulated transcripts are presented in red and downregulated transcripts in green (log2 fold change > 0.75). The genes affected upon exposure to SWCNTs (n = 52) and GO (n = 7) did not overlap. (**b**) Canonical pathways modulated in HMDM after exposure to SWCNTs (see Supplementary Table 2); ranking was performed according to multiple testing corrected p-value.

**Figure 2:**
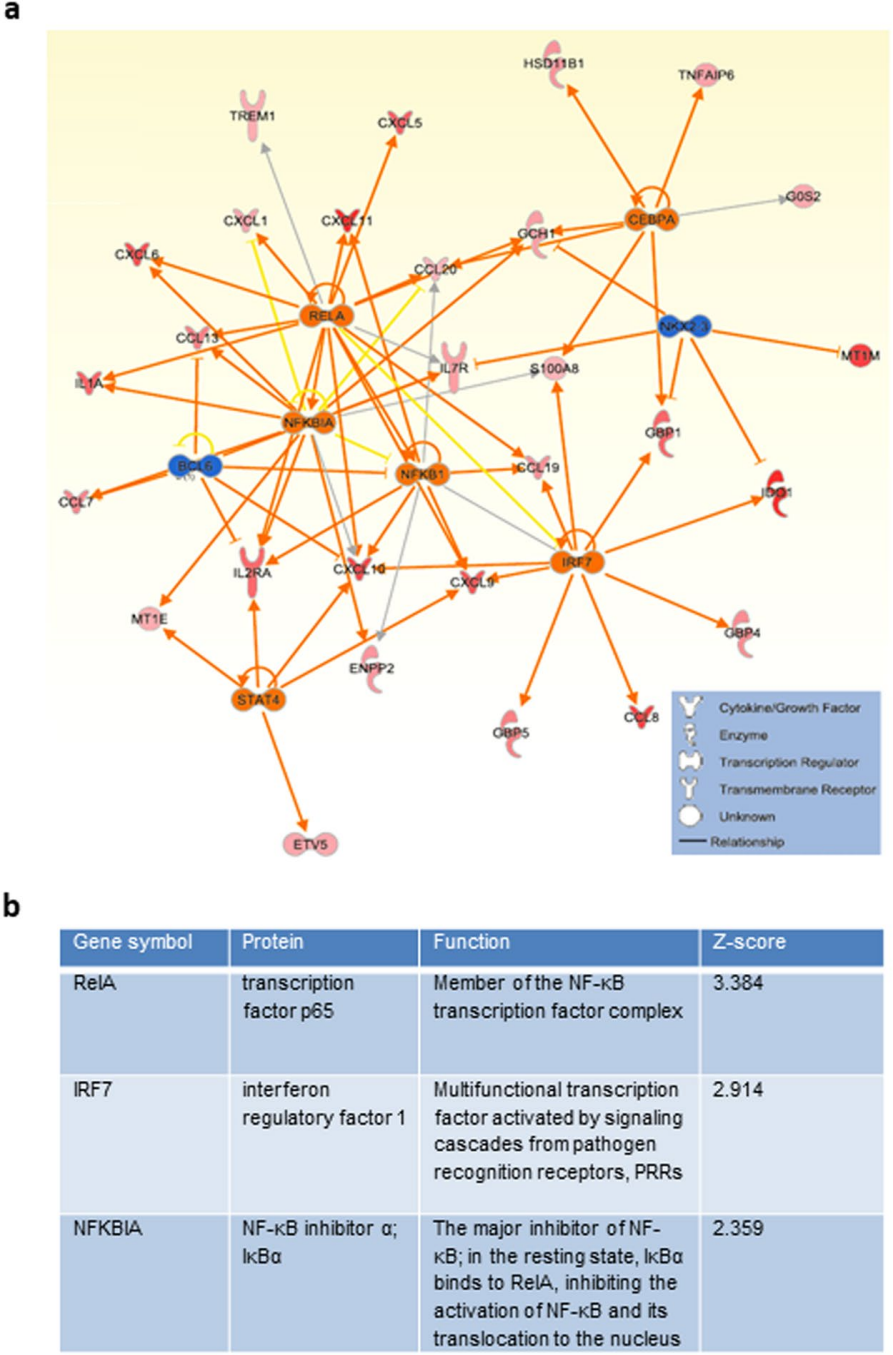
Upstream regulator analysis of the transcriptomics results. (**a**) The NF-κB network was identified as a potential upstream regulator of SWCNT-triggered responses in HMDM according to upstream regulator analysis (p < 0.01; Z-score > 2 S.D.). (**b**) Upstream regulator analysis^73^ of the data identified the modulation of NF-*κ*B network members in HMDM exposed to SWCNTs for 24 h. Data were analyzed through the use of IPA (QIAGEN Inc., www.qiagenbioinformatics.com/products/ingenuity-pathway-analysis).

To further evaluate the impact of the two nanomaterials on macrophages, we performed transcriptomics analyses following exposure for 6 h or 24 h to 10 or 30 µg/ml (*i.e*., sub-cytotoxic doses) of SWCNT or GO. Affymetrix^®^ GeneChip® Human Genome U219 arrays were employed for global assessment of gene expression. The microarray data have been submitted to the Gene Expression Omnibus Database (GEO accession no. GSE83516). Few differentially expressed genes were noted at 6 h (data not shown) and we therefore focused our analysis on the 24 h exposure time-point. The nanomaterial exposure at the latter time-point resulted in a specific transcriptional response with 52 differentially expressed genes in response to SWCNTs, while 7 genes were differentially expressed in response to GO (multigroup analysis by two-way ANOVA, p < 0.05, > 1.5-fold change compared to controls) (Fig. 1a, and Supplementary Table 1). Interestingly, the differentially expressed genes showed no overlap between the two nanomaterials. Subsequent canonical pathway enrichment analysis of differentially expressed genes using the Molecular Signatures Database showed that the three biological pathways with the most significant enrichment were those involved in *cytokine-cytokine receptor interaction*, *chemokine signaling pathway* and *chemokine receptor binding pathway* (multiple testing corrected p-values lower than 10^−25^) (Fig. 1b, and Supplementary Table 2). In addition, our analysis showed enrichment of the NF-*κ*B pathway (comprising 7 genes, q-value 10^−5.7^). Indeed, analysis of transcriptional regulation networks showed that NF-*κ*B was a central network connecting upregulated chemokines, and, moreover, that several members and targets of the NF-*κ*B pathway were significantly modulated in response to SWCNTs (Fig. 2a). Furthermore, upstream regulator analysis using the Ingenuity Pathway Analysis (IPA) software showed that the most significantly modulated NF-*κ*B pathway network members were RELA (p65), IRF7 and NFKBIA (IκBα) (Z-scores 3.4, 2.9 and 2.4, respectively) (Fig. 2b). Notably, according to a recent bioinformatics study, RELA and NFKBIA are both among the five key genes mediating NF-*κ*B pathway-related inflammatory responses and macrophage activation^20^. Taken together, the transcriptomics analysis suggested that chemokine signaling pathways are prominently deregulated by SWCNTs, but not by GO, and that NF-*κ*B is a potential upstream regulator of the transcriptional responses to SWCNTs.

### Validating the transcriptomics results

**Figure 3:**
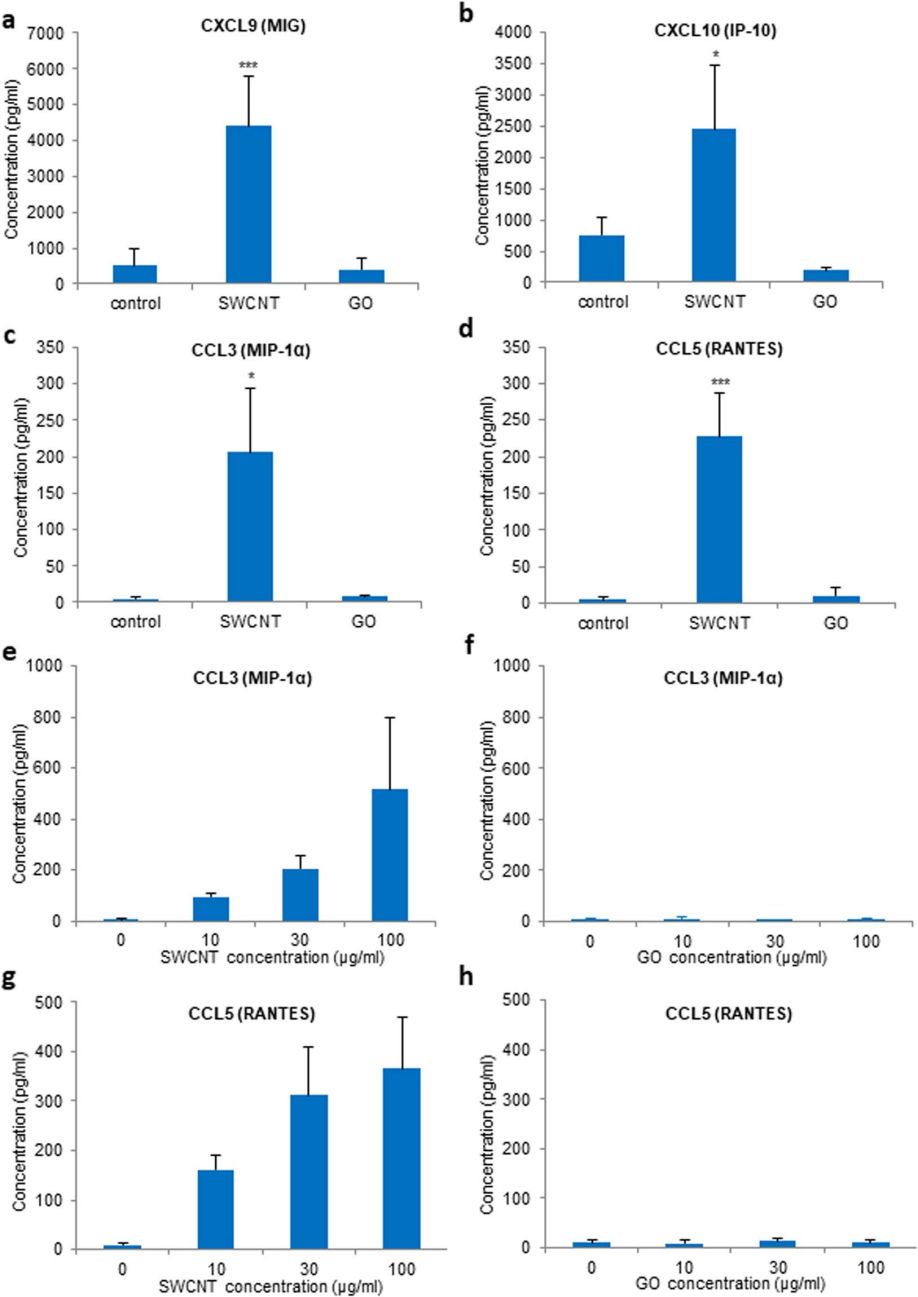
SWCNTs, but not GO, trigger macrophage secretion of chemokines. Secretion of chemokines by primary human macrophages (HMDM) after a 24 h exposure to SWCNTs or GO as measured by a multi-plex immunoassay. (**a**–**d**) Exposure of HMDM to 30 µg/mL SWCNTs showed a significant increase in CXCL9, CXCL10, CCL3/MIP-1α, and CCL5/RANTES, while there was no response in cells exposed to GO at the same concentration. (**e**,**g**) Dose-dependent secretion of CCL3/MIP-1α, and CCL5/RANTES in cells exposed to SWCNTs, while there was no response to GO at any of the concentrations tested (**f**,**h**). Data are shown as mean values ± S.D. of three independent experiments using cells from different donors; p-values by Student’s *t*-test, * < 0.05; *** < 0.001.

To validate these results, we assessed for macrophage production of chemokines. To this end, a multiplexed immunoassay for the detection of a defined set of cytokines/chemokines following exposure to SWCNTs or GO (10–100 µg/mL) was applied. We focused the analysis on four chemokines (CCL3/MIP-1α, CCL5/RANTES, CXCL9 and CXCL10) based on the significant upregulation of the corresponding genes according to our transcriptomics analysis (above). As shown in Fig. 3a–d, SWCNT-exposed cells produced high levels of all four chemokines, while GO-exposed cells failed to secrete these chemokines, which is thus in accordance with the microarray results. The secretion of CCL3 and CCL5 in SWCNT-exposed HMDM was dose-dependent (Fig. 3e–h). These data thus corroborated the transcriptomics results. To further control for any potential endotoxin contamination, the CNTs were subjected to calcination at 250 °C to remove any residual endotoxins. The samples were recharacterized (in cell culture medium) and shown to display similar ζ-potential values while DLS measurements showed no significant changes (i.e., no agglomeration) (Fig. S4a,b). We then monitored the production of CCL5 in macrophages following exposure for 12 h to calcined SWCNTs (30 µg/mL) and found that the SWCNTs were still capable of triggering CCL5; moreover, this was not affected by polymyxin B, indicating that the observed effect is intrinsic to the SWCNTs and not a result of microbial contamination (Fig. S5a).

### Dissecting the signaling pathway

**Figure 4:**
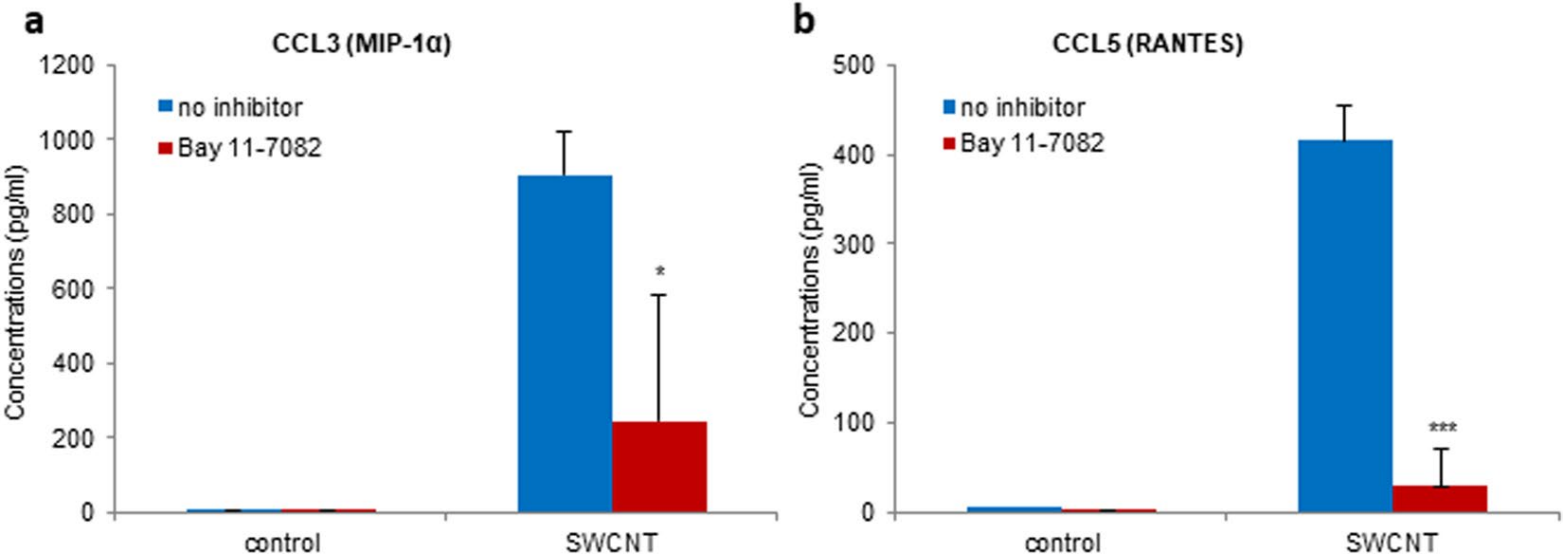
SWCNT-triggered chemokine secretion is NF-κB-dependent. Pretreatment with the NF-κB inhibitor, Bay 11-7082 (10 µM) of HMDM exposed to 30 µg/mL SWCNTs or medium alone reduced the secretion of CCL3 (**a**) and completely blocked the secretion of CCL5 (**b**), by multi-plex assay. Data are mean values ± S.D. of three independent experiments using cells from different donors; p-values by Student’s *t*-test, * < 0.05; *** < 0.001.

As noted above, upstream regulator analysis of the microarray data indicated that the NF-*κ*B signaling pathway was involved in the transcriptional regulation of chemokine expression by SWCNTs. To validate this *in silico* prediction, HMDM were pretreated with the NF-*κ*B inhibitor, Bay 11-7082, and chemokine secretion in response to SWCNT exposure was determined. SWCNT-induced production of CCL3 (Fig. 4a) was significantly reduced upon preincubation with Bay 11-7082 (10 µM) and the secretion of CCL5 was completely blocked by the inhibitor (Fig. 4b), thus confirming a role for NF-*κ*B. Next we aimed to address how NF-*κ*B is activated by SWCNTs.

**Figure 5:**
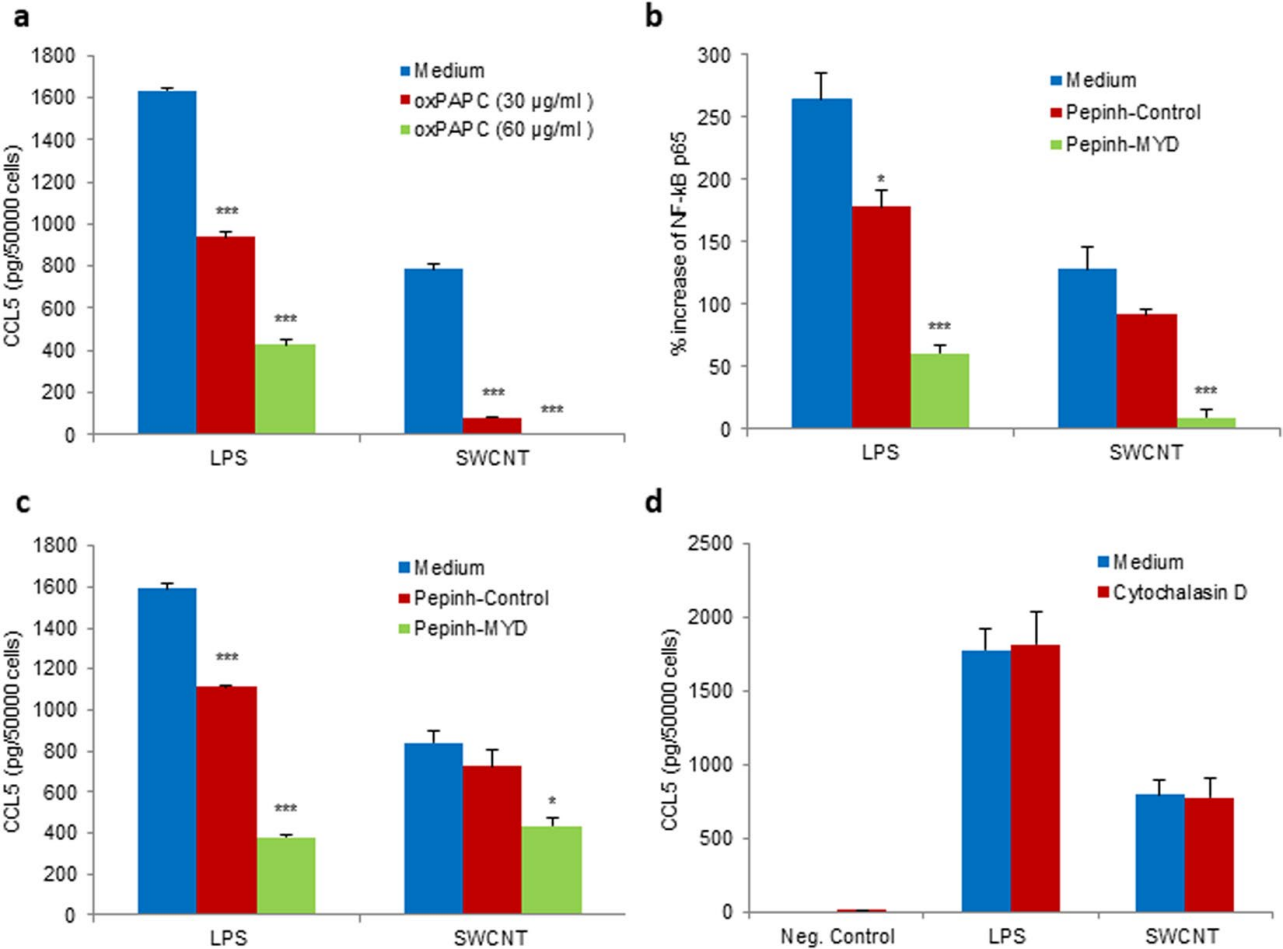
TLR2/4- and MyD88-dependent secretion of chemokines. (**a**) Significant reduction of CCL5 secretion in HMDM after 12 h exposure to SWCNT (30 µg/mL) in the presence of the TLR2/4 inhibitor, oxPAPC (30 or 60 µg/mL). oxPAPC also blocked CCL5 secretion triggered by LPS (0.1 µg/mL) in a dose-dependent manner. Furthermore, the MyD88 inhibitor, Pephinh-MYD (25 µM), but not Pepinh-Control (25 µM), reduced NF-kB p65 phosphorylation (**b**) and CCL5 expression (**c**) in cells exposed to SWCNT (30 µg/mL) for 12 h. Pephinh-MYD (25 µM) also reduced NF-kB activation and CCL5 secretion by LPS (0.1 µg/mL). NF-kB p65 phosphorylation and CCL5 expression was determined by ELISA. (**d**) Cytochalasin D (10 μM), an inhibitor of actin polymerization, does not affect CCL5 secretion in HMDM exposed for 12 h to SWCNT (30 µg/mL). LPS (0.1 µg/mL) was included as a control. CCL5 levels were determined by ELISA. Data shown in panels **a** to **d** are reported as mean values ± S.D. of at least three independent experiments using cells from different donors. p* < 0.05; ** < 0.01; *** < 0.001 (one-way ANOVA with post-hoc tukey’s test).

The recognition of so-called PAMPs by different families of evolutionarily conserved PRRs (PRRs) initiates a signaling cascade that leads to the transcription of inflammatory cytokines and chemokines to eliminate pathogens and attract other immune cells to the site of infection^21^. In particular, Toll-like receptors (TLRs) play a key role in innate immunity^16^. TLRs activate multiple signaling pathways by recruiting adaptor proteins, such as myeloid differentiation factor 88 (MyD88), which initiate signal transduction pathways that culminate in the activation of transcription factors, *eg*., NF-*κ*B, with ensuing cytokine/chemokine production. TLR4 is a receptor for bacterial LPS while TLR2 has specificity for multiple microbial components derived from bacteria, fungi, viruses, and mycoplasma^21^. The oxidized phospholipid, 1-palmitoyl-2-arachidonyl-sn-glycero-3-phosphorylcholine (oxPAPC) is known to inhibit LPS signaling *via* TLR2 and TLR4^22^. To assess whether SWCNTs are capable of activating NF-*κ*B *via* TLRs, we preincubated HMDM with oxPAPC (30 or 60 µg/mL) prior to exposure to SWCNTs (30 µg/mL). LPS (100 ng/mL) was included as a positive control. As shown in Fig. 5a, oxPAPC significantly reduced LPS-induced secretion of CCL5. Moreover, SWCNT-induced production of CCL5 was completely blocked. Next, we tested whether inhibition of the adaptor protein, MyD88 would suppress NF-*κ*B activation. To this end, cells were preincubated with Pepinh-MYD, a 26 aa peptide that blocks MyD88 signaling by inhibiting its homodimerization^23^. Pepinh-Control, a control peptide, was included as a negative control. Pepinh-MYD (25 µM) significantly reduced LPS-induced NF-*κ*B activation, as determined by the quantification of p65 phosphorylation, and SWCNT-induced activation of NF-*κ*B was also blocked by Pepinh-MYD, but not by Pepinh-Control (25 µM) (Fig. 5b). Moreover, inhibition of MyD88 impeded chemokine production in cells exposed to SWCNTs. Thus, Pepinh-MYD (25 µM) significantly reduced LPS-induced CCL5 secretion, and SWCNT-triggered release of CCL5 was also reduced by Pepinh-MYD, but not by the control peptide (Fig. 5c). These data thus provided evidence for TLR2/4-MyD88-NF-*κ*B signaling in SWCNT-induced chemokine production in human macrophages.

SWCNTs are internalized by HMDM at 24 h (Fig. S2) and we recently provided evidence that macrophage uptake of SWCNTs may occur already after a few hours^24^. To assess whether cellular uptake of SWCNTs is required for chemokine responses, we determined the secretion of CCL5 in HMDM exposed to SWCNTs (30 µg/mL) following preincubation with or without cytochalasin D (10 µM), an inhibitor of actin polymerization that blocks endocytosis^24^. As shown in Fig. 5d, cytochalasin D did not affect LPS-induced or SWCNT-induced production of CCL5, suggesting that this event is relayed *via* cell surface signaling. We also cultivated macrophages in medium with or without 10% FBS in order to test whether the effect of SWCNTs on chemokine secretion was influenced by the presence of serum proteins. As shown in Fig. S5b, SWCNT-triggered production of CCL5 was comparable in the presence or absence of serum. LPS was included as a positive control.

**Figure 6:**
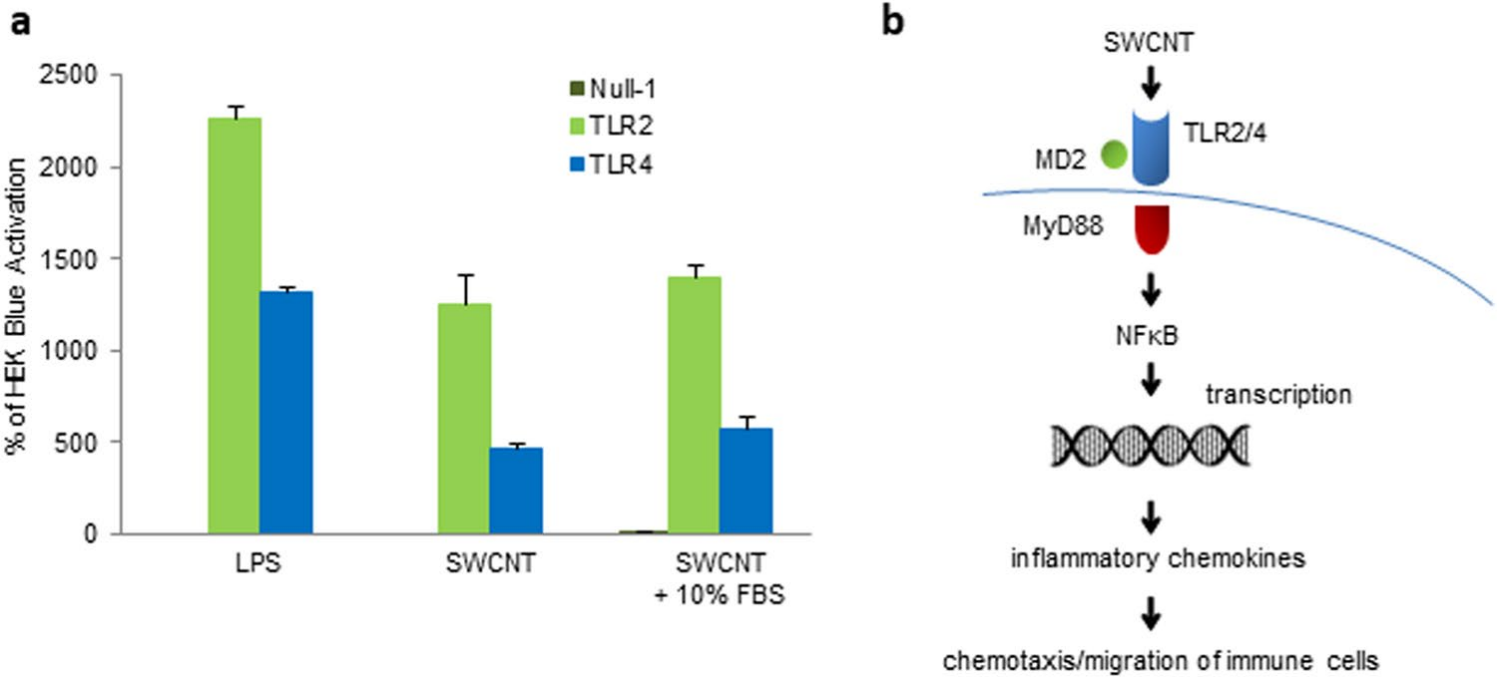
SWCNTs trigger TLR2 and TLR4 activation. (**a**) HEK 293 cells co-transfected with human TLR2 (HEK-Blue™ hTLR2) or TLR4 (HEK-Blue™ hTLR4 cells) and an NF-κB/AP-1-secreted embryonic alkaline phosphatase (SEAP) reporter gene were exposed to SWCNT (30 µg/mL) for 12 h in the presence or absence of 10% FBS. LPS (0.1 µg/mL) was included as a positive control. SWCNTs activated TLR2/4 independently of the presence of serum in the culture medium. Data are shown as mean values ± S.D. of three independent experiments. (**b**) Schematic diagram showing the ‘sensing’ of SWCNTs by HMDMs *via* TLR receptors resulting in MyD88-dependent activation of NF-kB leading to nuclear translocation of NF-kB with transcription and secretion of CCL5. The secreted chemokine(s) induce chemotaxis of immune cells bearing the corresponding receptor(s).

To confirm the findings obtained in monocyte-derived macrophages, and in order to address the role, if any, of specific TLRs for the ‘sensing’ of SWCNTs, we used HEK293 cells stably transfected with human TLR2 or TLR4 and an NF-*κ*B-inducible reporter gene^25^. Furthermore, in order to ascertain whether SWCNTs are capable of TLR activation *per se*, or whether the interaction is due to serum proteins adsorbed on the surface of the nanomaterials^26^, we performed the experiments in reporter cell lines cultured in medium supplemented or not with 10% fetal bovine serum (FBS). LPS (100 ng/mL) was used as a positive control. LPS triggered pronounced activation of TLR4 and a significant activation of TLR2 (Fig. 6a). Interestingly, SWCNTs also activated TLR4 and, to a lesser degree, TLR2, and the level of activation in the presence and absence of FBS was indistinguishable. The latter finding suggested that SWCNTs are sensed directly by TLRs (Fig. 6b).

### Functional role of chemokine secretion

**Figure 7:**
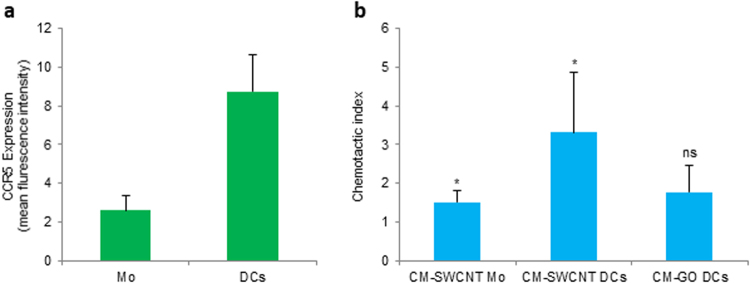
Macrophage secreted factors promote migration of DCs. (**a**) Expression of the chemokine receptor CCR5 in primary human monocytes (Mo) and monocyte-derived dendritic cells (DCs) was determined by flow cytometry. The average expression of CCR5 in cells from three different donors is depicted. (**b**) Migration of monocytes (Mo) and DCs in response to conditioned medium (CM) of human monocyte-derived macrophages (HMDM) exposed to 100 µg/mL SWCNT (CM-SWCNT) or 100 µg/mL GO (CM-GO). Cell migration (3 h period) was determined by using transwell chemotaxis microchambers. Data are shown as mean values ± S.D. of three independent experiments; p* < 0.05, Student’s *t*-test.

Chemokines (Greek: *kinos*, movement) are named for their role in inducing directed migration or chemotaxis in neighboring responsive cells. We therefore addressed whether the conditioned medium of macrophages exposed to SWCNT *versus* GO would act as a chemoattractant for other immune cells. Considering that CCL3 and CCL5 are the most effective chemoattractants for immature DCs^27^, we studied cell migration using primary human monocyte-derived DCs *versus* non-differentiated primary human monocytes. Surface expression of the chemokine receptor, CCR5 was higher for DCs than monocytes (Fig. 7a), in line with published results^28^. Furthermore, conditioned medium of SWCNT-exposed HMDM was a weak chemoattractant for monocytes, but promoted the migration of DCs (p < 0.05) (Fig. 7b). Thus, the conditioned medium served as a chemoattractant for cells expressing CCR5 and did not remarkably affect the migration of cells that expressed low levels of CCR5. However, conditioned medium of GO-exposed HMDM did not promote cell migration (Fig. 7b), in line with the observation that only SWCNTs triggered chemokine secretion.

### Molecular docking studies

**Figure 8:**
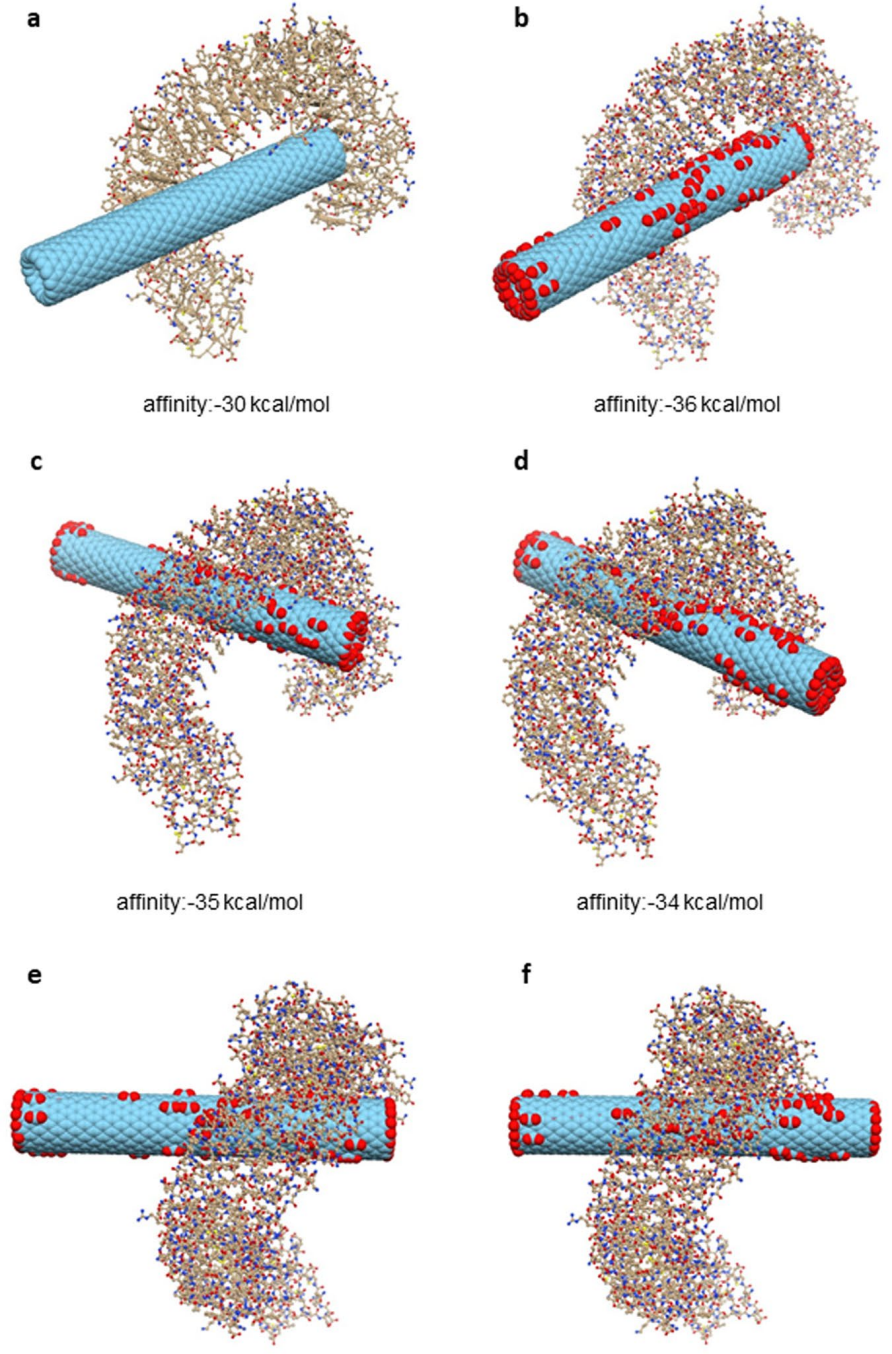
Molecular docking of CNTs and TLR4. Results of docking simulations of pristine and carboxylated CNTs and TLR4. (**a**) The best binding mode for pristine CNT. (**b**) best binding mode for carboxylated CNT. (**c**) lateral view of the second top binding pose for carboxylated CNT. (**d**) lateral view of the third top binding pose for the same CNT. (**e**) top view of the same configuration as in (**c**). (**f**) top view of the same configuration as in (**d**). The mechanism involves interactions of the target protein with both the tip and side-wall of CNTs.

**Figure 9:**
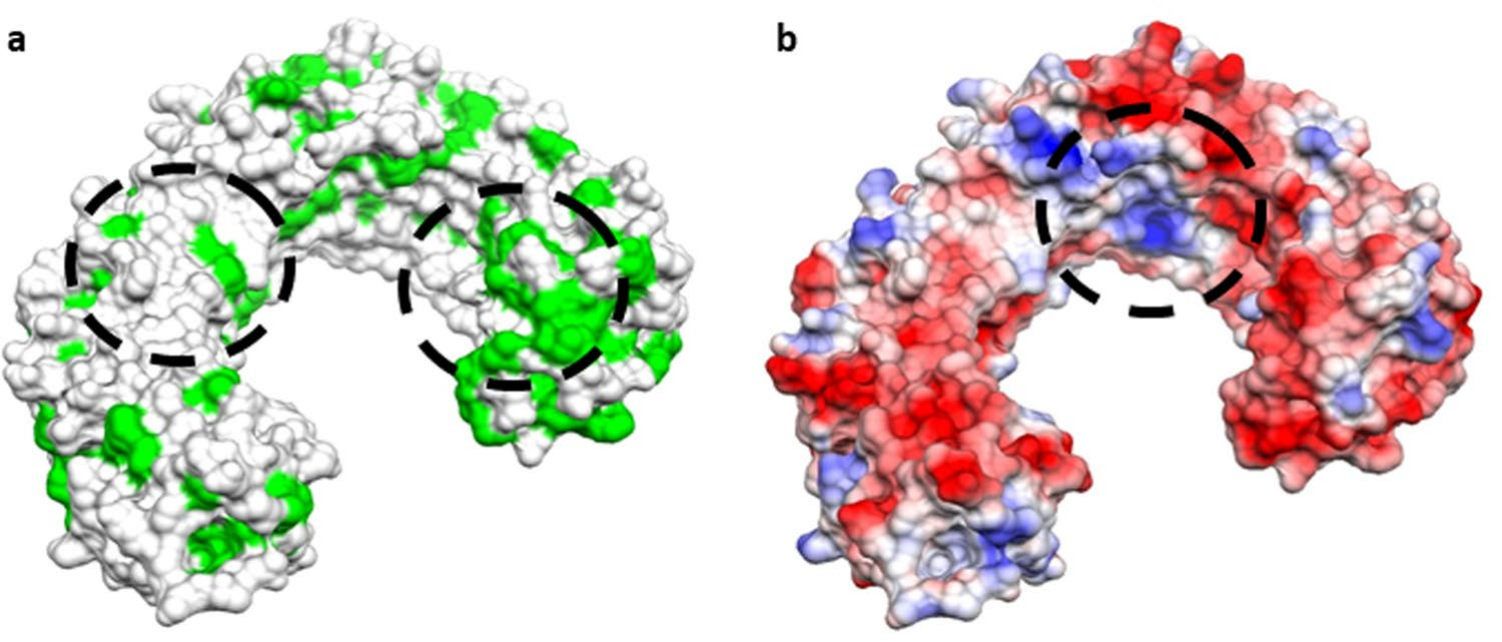
Identification of interaction sites. The molecular surface of TLR4 is shown with two different coloring schemes: (**a**) hydrophobicity scale (hydrophobic residues are depicted in green) and (**b**) electrostatic potential (coloring scale runs from red to blue in the interval of values [−16.89:16.89] kT/e). Circled areas represent the regions of TLR4 that directly interact with CNTs by hydrophobic contact (**a**) and by electrostatic interaction (**b**).

Previous theoretical studies have suggested that C_60_ fullerenes and CNTs may block K^+^ channels^29,30^. In addition, recent computational studies suggested that the internal hydrophobic pockets of some TLRs might be capable of binding carbon-based nanostructures^31^. We performed molecular modelling of the TLR4:CNT complex to further elucidate how TLRs can interact with the CNTs; the studies were done in the absence of a protein corona. To better reproduce the experimental conditions, both pristine and carboxylated CNTs were modelled. The O:C atom ratio corresponding to the experimental zeta potential was set to 0.15^32^, which resulted in 84 carboxyl groups. Docking simulations of pristine CNTs gave rise to a unique cluster of very similar poses within the pre-defined energy range (10 kcal/mol). Conversely, carboxylated CNTs showed diverse binding modes, with interaction free energies differing only slightly (~1 kcal/mol). The best scoring binding mode, observed in both pristine and carboxylated CNTs, revealed two regions in TLR4, one interacting with the tip of the CNT and another in contact with its side-walls (Fig. 8a,b). While the first region is localized in a highly hydrophobic area, which encompasses residues from Ile108 to Asn265, the second is found in the loops around Ile412 and Leu434 (Fig. 9a,b). In oxidized CNTs, His159 and Arg264 are also within a distance to the carboxyl groups that is compatible with salt bridge formation. Alternatively, in the second region, Arg382, His431 and His458 are close enough to form ion-pair interactions with carboxyl groups on the CNT side-walls. Overall, the best binding mode is essentially guided by hydrophobic contacts between TLR4 and CNTs, but in the case of carboxylated CNTs the intermolecular interaction is strengthened by short-range electrostatics. The second and third top binding modes of carboxylated CNTs correspond to a completely different configuration, in which side-walls are in close contact with a large portion (from residue 87 to 289) of the TLR parallel beta-sheet pattern in the inner part of the protein (Fig. 8c–f, Fig. 9a,b). Interestingly, the area of interaction of these poses intersects one of the two regions already observed for the best pose. This finding is consistent with the negligible energy difference of these binding modes with respect to the best scoring mode. Nevertheless, as opposed to the latter, these two classes of poses are clearly dominated by electrostatic interactions, as shown by the distances between the carboxyl groups and some charged residues (His179, Arg257, Arg289, Arg355), which are compatible with the presence of stabilizing ion-pair interactions.

## Discussion

The innate immune system does not respond to microbes in a nonspecific manner; in fact, pathogen recognition by the innate immune system is specific, relying on PRRs that have evolved to detect molecular signatures known as PAMPs^21^. Thus, a relatively small number of immune receptors are employed by macrophages and other immune cells to detect a vast array of microorganisms; it is intriguing to speculate that similar principles or mechanisms might be deployed for immune recognition of various classes of nanoparticles^33^. However, to date, there are few if any examples of specific immune sensing of engineered nanomaterials and the problem is confounded by a number of factors. First, many studies are performed using nanoparticles that are not well characterized, or not uniform in appearance within the same sample, making it difficult to draw conclusions regarding specific properties of nanoparticles and their biological behavior^34^. In addition, nanoparticles are frequently contaminated with bacterial endotoxin, as may be the case for other biomaterials, leading potentially to erroneous results, especially when studying interactions with immune-competent cells^17^. Furthermore, nanoparticles are known to rapidly adsorb proteins and other biomolecules, and this is thought to endow the nanoparticles with a new, biological ‘identity’ such that these adsorbed biomolecules could dictate biological interactions: cells may not ‘see’ the pristine nanoparticle surfaces^35^. In addition to these considerations, it has been argued that there are no nano-specific (*i.e*., size-dependent) biological effects of nanoparticles, and therefore no novel effects are to be expected^36^. However, it is worth noting that many biological processes transpire at the nano-scale. Thus, it follows from this argument that nanoparticles, as a function of their small size, may interfere with biological processes in a manner not seen for larger particles^37^. Park *et al*.^29^ showed that purified SWCNTs blocked K^+^ channel subunits in a dose-dependent manner, presumably by ‘plugging’ the channel, by virtue of the small (nano-scale) diameter of the CNTs. Similarly, theoretical studies of C_60_ fullerenes have indicated that K^+^ channels are blocked by fullerenes through a mechanism that is governed purely by shape complementarity^30^. Moreover, C_60_ fullerenes were found to interact with and modulate the function of the Ca^2+^/calmodulin-dependent protein kinase II (CaMKII), a serine/threonine kinase central to Ca^2+^ signal transduction^38^. The latter study provides a compelling example of an inorganic nanoparticle that acts like a cellular signaling protein, or protein ‘mimic’. The present study suggests that SWCNTs may interact with specific PRRs on the cell surface, leading to chemokine secretion. Thus, the response to SWCNTs apparently mimics the immune response to pathogens. Interestingly, it was suggested previously that nanoparticle-induced dysregulation of macrophage responses could be due to shared uptake pathways for nanoparticles and bacteria; however, whether or not this was mediated *via* direct receptor binding of the nanoparticles was not disclosed^39^. Our results have shown that endotoxin-free SWCNTs and GO are taken up by primary human macrophages, but only the SWCNTs triggered chemokine production, and we also noted that chemokine production did not depend on cellular internalization of the SWCNTs. Using global gene expression analyses, we could show that SWCNTs induced transcriptional upregulation primarily of chemokine signaling pathways and these observations were borne out at the protein level. We could also validate the *in silico* prediction that the transcription factor, NF-*κ*B was an important regulator of chemokine production in SWCNT-exposed cells. Furthermore, our studies provided evidence for a role of the TLR2/4-MyD88-NF-*κ*B signaling pathway in SWCNT-induced chemokine production in macrophages (Fig. 6b). Indeed, using specific reporter cell lines, we demonstrated that SWCNTs *per se* are capable of activating TLRs, in the absence of a protein corona. This is the first study to show that SWCNTs can trigger TLRs and the findings thus provide some support for the proposal that ‘nanoparticle-associated molecular patterns’ or NAMPs could serve as ligands for PRRs on immune cells^40^. Quantum dots were previously shown to trigger chemokine expression *via* MyD88-dependent TLR signaling pathways, either at the cell surface or inside the cells, but whether or not the particles were directly ‘sensed’ by TLRs was not disclosed^41^. Furthermore, DCs were shown to respond to various synthetic biomaterials (polymers) through MyD88/TLR-dependent pathways, possibly through recognition of certain hydrophobic amino acids in the adsorbed proteins, or through a direct ‘recognition’ of hydrophobic material surfaces^42^. Remarkably, as the present study was under way, computational results suggesting that the internal hydrophobic pockets of some TLRs might be capable of binding carbon-based nanostructures, such as SWCNTs and C_60_ fullerenes were reported^31^. The latter theoretical studies are in line with the experimental data reported here, and are also concordant with our own molecular docking studies, as we shall discuss in more detail below. Taken together, TLR-dependent signaling could constitute a mechanism by which nanomaterials trigger inflammation. The present results also suggest that size-dependent, receptor-mediated effects may underlie some aspects of the toxicity of carbon-based nanomaterials^37^. However, if these effects are understood, immune activation by nanomaterials may also be harnessed for biomedical applications^43^.

One may ask why GO did not trigger chemokine production, or cell death, in HMDM, in particular in light of recent studies suggesting a role for TLR signaling in GO-induced cell death^44^. Aside from technical, yet non-trivial explanations, including the possibility of endotoxin contamination, it is also conceivable that GO could trigger cellular responses through a different mechanism: not *via* specific ligand-receptor interactions, but instead through a direct effect on the cell membrane, as shown previously for silica and uric acid crystals^45^. Indeed, the fact that GO sheets with large lateral dimensions show stronger effects on macrophages when compared to smaller GO sheets appears to argue against a specific receptor interaction and in favor of a non-specific membrane binding or masking effect^46^. Furthermore, previous studies using primary human macrophages have shown that graphene and CNTs differ significantly in their uptake mechanisms^47^. We entertained the possibility that the differences between SWCNTs and GO could be explained by differences in the adsorbed layer of serum proteins (or, bio-corona), although our characterization results showed that both nanomaterials have a near-identical surface charge. However, even if the composition of the adsorbed protein layer were found to be similar for SWCNTs and GO (this remains to be studied), the manner in which the proteins are displayed on the surface could also impact on subsequent cellular responses. Hence, protein display on a flat surface as in the case of GO may differ from the presentation of the same protein(s) on SWCNTs with a diameter of a few nm and a more pronounced surface curvature. Interestingly, recent studies have shown that the secondary structure of the corona proteins, exemplified by serum albumin, determines cell surface receptor usage by polystyrene nanoparticles^48^. However, in the present study, a role for adsorbed proteins was apparently excluded insofar as the experiments using reporter cell lines showed a similar degree of TLR2 and TLR4 activation in the presence and absence of FBS. In addition, SWCNT-triggered CCL5 secretion by primary macrophages was not influenced by the presence or absence of serum. Thus, while it is clear that nanomaterials may adsorb proteins when incubated in FBS-containing cell culture medium^49^, and while this layer of proteins may impact on cell uptake or cytotoxicity, this does not mean that the material itself is unavailable for interactions with cell surface receptors. Recent work has shown that the protein corona composition on the surface of nanoparticles changes with increasing plasma concentrations^50,51^, and it was suggested that while the surface is “relatively well covered” when nanoparticles are immersed in 10% plasma, the so-called ‘hard’ corona continues to evolve at increasing concentrations of plasma^50^. In addition, in a previous study of dextran-coated superparamagnetic iron oxide, Simberg *et al*.^52^ reported that both the dextran coating and the iron oxide core remained accessible to specific probes after incubation in plasma, suggesting that the nanoparticle surface could be available for recognition by macrophages, regardless of protein coating. To further address if and how SWCNT are able to interact directly with TLRs, we performed molecular docking simulations of model nanotubes and TLR4. Overall, these studies suggested two potential mechanisms of interactions between CNTs and TLR4. While the first, shared by both pristine and carboxylated CNTs, involves interactions of the target protein with both the tip and the side-wall of CNT, the second class of binding modes, peculiar for carboxylated CNT, requires only interactions along the carboxylated side-walls. For this reason, at least in principle, only the second class of mechanisms seems to be compatible with long CNTs. Moreover, the second class of poses is formed by interactions localized in an area with longitudinal length of about 3.5 nm, which is a rather small portion of the overall length of the CNTs that were studied experimentally. This characteristic makes the binding to TLR4 compatible with CNTs partially bound to serum proteins. Indeed, the efficiency of the protein corona binding is well-known to be proportional to the radial dimensions of the CNT^53–55^. Thus, the small diameter of the CNTs may hinder the binding of serum proteins and limit the portion of surface effectively covered by corona, hence leaving enough space for the direct interaction between the CNT and TLR4. In addition, it cannot be excluded that the tips of the CNTs are spared from coating with serum proteins, thus allowing the CNTs to engage with receptors tip-first.

Chemokines are a family of secreted signaling proteins that induce chemotaxis (migration) of responsive cells bearing the corresponding receptor(s). In the present study, we found that CCL3 and CCL5, along with several other chemokines, were upregulated and secreted by macrophages exposed to SWCNTs, but not GO. CCL3 and CCL5 both bind to CCR5, a receptor that is expressed by T cells, macrophages and DCs and plays a pivotal role in inflammatory responses to infections^56^. The current observation that conditioned medium of SWCNT-exposed macrophage cultures – but not GO-exposed cultures – promoted migration of DCs suggests a mechanism for the recruitment of immune cells into the lungs following exposure to SWCNTs. Pulmonary exposure to SWCNTs is known to result in the accumulation of different types of immune cells, including neutrophils, eosinophils and macrophages^57^, much like an infection. In addition, and more directly linked to the current *ex vivo* results, we demonstrated previously that pharyngeal aspiration of SWCNTs induced the infiltration of antigen-presenting DCs into the lungs of mice^7^. Further to this point, it is pertinent to ask whether the current results obtained using isolated human macrophages show any correlation to published *in vivo* results on CNTs. Given the fact that chemokine signaling pathways were found to be among the most prominently affected pathways at the transcriptional level, one may ask whether this aligns with the *in vivo* effects of SWCNTs or of related nanomaterials. Indeed, this seems to be the case. In a recent study, the resolution of inflammatory responses was shown to be delayed in CCR5 knockout mice exposed to SWCNTs when compared to WT mice^58^, pointing to a key role of CCR5 and its corresponding ligand(s) for immune responses to SWCNTs. As already mentioned, CCL3/MIP-1α and CCL5/RANTES, along with CCL4/MIP-1β, are known ligands of CCR5, and CCR5 plays a pivotal role in inflammatory responses to infections^56^. These observations suggest, again, that SWCNTs are sensed as pathogens by the immune system. This also means that conserved mechanisms and pathways are elicited in response to nanomaterials. Thus, while biological responses to nanomaterials may be size-dependent, this does not necessarily mean that the responses are ‘novel’. This, therefore, suggests that attempts to mitigate the adverse effects of such nanomaterials can be focused on well-known and conserved pathways. Furthermore, using a cell-specific depletion and repopulation approach in mice, Frank *et al*.^59^ could show that MyD88 mediated the effector functions of alveolar macrophages (AMs) in the acute inflammatory responses to MWCNTs. Hence, MyD88 inhibition in donor AMs abrogated their capacity to reconstitute MWCNT-induced inflammation when adoptively transferred into AM-depleted mice. The latter *in vivo* results are in line with the present *in vitro* studies showing a role for MyD88/TLR-dependent signaling in human macrophages in response to SWCNTs. Moreover, several gene expression studies have been published showing that chemokine signaling pathways are upregulated *in vivo* in the lungs of animals (mice or rats) exposed to CNTs, thus lending further support to the validity of the present findings. Fujita *et al*.^60^ reported that a single intratracheal instillation of SWCNTs in rats elicited upregulation of a large number of genes involved in inflammatory responses until 90 or 180 days post-instillation including several genes encoding for chemokines, among them CCL3/MIP-1α. More recently, Kinaret *et al*.^61^ reported that genes involved in chemokine signaling and cytokine-cytokine receptor interaction pathways were significantly upregulated following exposure of mice to MWCNTs, using two alternative airway exposure procedures, pharyngeal aspiration (a single dose per day for 4 days) and inhalation (4 h per day for 4 days) with collection of samples after 24 h; interestingly, both procedures elicited a very similar inflammatory response. Additionally, we have recently conducted a study in which mice were exposed twice a week for 3 weeks to SWCNTs through pharyngeal aspiration followed by collection of samples at several different time-points. Detailed analyses of the microarray samples obtained at different time-points post-exposure from different tissues including the lungs will be reported elsewhere (Tuomela *et al*., manuscript in preparation), but we noted that the pathways affected by SWCNTs *in vivo* overlapped significantly with the pathways triggered in human macrophages. The analysis showed that a greater number of genes were differentially expressed in the lungs of exposed mice relative to HMDM, including several interleukins, cytokines and chemokines, and corresponding receptors, perhaps reflecting the fact that the lungs represent a more complex mix of different cell types. Indeed, it is likely that several different cell types, including neutrophils, eosinophils, macrophages and lung epithelial cells are involved in the orchestration of the inflammatory response to nanomaterials. Chen *et al*.^62^ concluded in a recent study that alveolar epithelial cells, and not macrophages, are the major producers of cytokines/chemokines in mice exposed to small, spherical carbon nanoparticles, and Katwa *et al*.^63^ showed that mast cells are required for certain pulmonary and cardiovascular responses to MWCNTs. On the other hand, other recent studies have unambiguously demonstrated that alveolar macrophages are major effector cells in MWCNT-induced acute inflammation^59^. Overall, a common feature, as evidenced from this survey of the literature, is that chemokine signaling pathways are prominently upregulated following pulmonary exposure to CNTs.

In sum, our study showed that SWCNTs induce chemokine secretion in macrophages through a TLR2/4-MyD88-NF-*κ*B signaling pathway. GO, on the other hand, did not elicit chemokine responses. These studies shed light on the interactions of nanomaterials with the immune system and suggest that nanomaterials might be sensed as pathogens or NAMPs.

## Methods

### Reagents

Lipopolysaccharide (LPS) (*E. coli* serotype O111:B4), polymyxin B, and cytochalasin D were obtained from Sigma-Aldrich. Bay 11-7082 was from Calbiochem. Pepinh-MYD, Pepinh-Control, and 1-palmitoyl-2-arachidonyl-sn-glycero-3-phosphorylcholine (oxPAPC) were all from InvivoGen (Toulouse, France). ZnO nanoparticles (ZincoxTM 10) were from IBU-Tec Advanced Materials AG (Weimar, Germany)^64^.

### Nanomaterial preparation

SWCNTs (CNI, Inc., Houston, TX) produced by the high pressure CO disproportionation process (HiPco) technique, employing CO in a continuous-flow gas phase as the carbon feedstock and FeCO_5_ as the iron-containing catalyst precursor, and purified by acid treatment to remove metal contaminates^65^, were used. GO was synthesized by a modified Hummer’s method, as previously described^66^. The synthesized GO was washed several times by a mixture of water and ethanol, and the obtained slurry was dialyzed for 3 days to exclude any contamination with cations. Both nanomaterials were dispersed in dH_2_O. Before use, the materials were diluted in cell culture medium to 100 µg/ml and sonicated in a water bath for 10 min (Branson 1510, 40 kHz).

### Nanomaterial characterization

The SWCNTs were previously characterized with respect to purity, surface area, size and shape^7^. The final dispersion comprised of 0.23 wt% iron according to inductively coupled plasma mass spectrometry (ICP-MS). The specific surface area measured by the nitrogen absorption–desorption technique (Brunauer Emmet Teller method, BET) was around 1040 m^2^/g. Transmission electron microscopy (TEM) revealed that the SWCNTs had a fibrous structure with an average diameter of 1–4 nm diameter and average length of 0.5–2 µm. SWCNTs. The ζ-potential was −42.3 ± 0.9 mV. For GO, TEM measurements and corresponding electron diffraction (ED) pattern were performed by dropping a GO dispersion on a carbon-covered standard TEM grid (QUANTIFOIL Multi A) and subsequent drying under air. Samples were analyzed on a ZEISS Leo912 transmission electron microscope operated at an acceleration voltage of 120 kV. TEM showed that GO had a two-dimensional structure with smooth and regularly shaped surface (Fig. S3a) with an average diameter of 1.1 ± 0.3 µm. The ζ-potential of GO flakes was recorded using Malvern Zetasizer Nano ZS and was found to be −42.0 ± 1.2 mV. The successful oxidation was proven by XRD which showed the shift of the (002) plane of graphite at 26.5° to 9.9° (022) of GO. Furthermore, FTIR analysis using a Perkin Elmer FT spectrometer between 400 and 4000 cm^−1^ revealed characteristic peaks at 3300, 1720, 1620, 1405, 1220, 1041 cm^−1^ which correspond to the alcohol-, ether- and carboxyl surface groups (Fig. S3b). The chemical composition of GO was determined by X-ray photoelectron spectroscopy (XPS) using a Surface Science Instruments ESCA M-Probe XPS spectrometer with a monochromatic Al K-α source of 1486.68 eV. Survey XPS spectra (Fig. S3c) were acquired with pass energy (PE) of 158.9 eV, 0.5 eV step size, 125 ms dwell time and averaged over 7 scans. The high resolution C1s and O1s XPS spectra (Fig. S3d,e) were acquired with PE of 22.9 eV, 0.05 eV step size, 175 ms dwell time and averaged over 25 scans. Spectra of the insulating samples were charge corrected by shifting all peaks to the adventitious carbon C 1 s spectral component binding energy set to 284.8 eV. CasaXPS software was used to process the spectra.

### Calcination of nanomaterials

SWCNT samples (0.7 mg/mL, 750 µL in dH_2_O) were placed into a 10 mL flask and dried under vacuum at 40 °C. After the solvent was removed, the atmosphere was changed to nitrogen and the sample was heated to 250 °C (first 200 °C and then 250 °C to avoid overheating). The sample was kept at 250 °C for 1 h, then cooled down and re-dispersed in distilled water. Then, SWCNTs were dispersed at 30 µg/mL in distilled water, PBS, DMEM medium, or DMEM medium supplemented with 10% FBS by ultrasonication for 10 min using a bath sonicator and size and zeta potential of the samples were measured using Zetasizer Nano ZS (Malvern Instruments Ltd., UK) as described^67^. For cellular interaction studies, calcined SWCNTs were dispersed in endotoxin-free water under sterile conditions in laminar flow. HMDM were then exposed to SWCNT (30 µg/mL) for 12 h in DMEM medium supplemented with 10% FBS in the presence or absence of polymyxin-B sulfate (10 µM). CCL5 production was measured using ELISA as described below.

### Endotoxin assessment

Endotoxin content in SWCNT and GO dispersions was assessed using the chromogenic limulus amoebocyte lysate (LAL) assay (Charles River Endosafe, Charleston, SC). The endotoxin content was found to be below FDA-mandated limits of acceptance (0.5 EU/mL) (data not shown). To verify these results, SWCNT samples were also assessed using the TNF-α expression test (TET) that enables unequivocal detection of endotoxin with a sensitivity that is comparable to the conventional LAL assay, but without any interference with the assay, as described previously^18^. In brief, HMDM were exposed to nanomaterials or lipopolysaccharide (LPS) in the presence or absence of the specific LPS inhibitor, polymyxin B (10 µM) and TNF-α secretion was measured at 24 h of exposure using a Human TNF-α ELISA Kit purchased from Abcam (Sweden).

### Primary human immune cells

Human mononuclear cells were isolated from buffy coats obtained from adult blood donors (Karolinska University Hospital, Stockholm, Sweden) by density-gradient centrifugation using Lymphoprep (Nycomed). Monocytes were separated based on CD14 expression using CD14 MicroBeads (Miltenyi Biotech Ltd) following the manufacturer’s instructions^68^. Cells were grown in RPMI-1640 medium supplemented with 10% heat-inactivated fetal bovine serum (FBS), 2 mM glutamine, 100 U/mL penicillin, and 100 μg/mL streptomycin (Gibco). For the generation of human monocyte-derived macrophages (HMDM), cells were cultured with 50 ng/mL recombinant macrophage colony-stimulating factor (M-CSF, R&D Systems) for 3 days. To confirm macrophage differentiation, surface expression of CD11b was determined using a PE-conjugated CD11b antibody (BD Biosciences) and measured using a BD LSRFORTESSA flow cytometer (Fig. S6). For the generation of dendritic cells (DCs), CD14^+^ cells were instead cultured with 10 ng/ml granulocyte-macrophage colony-stimulating factor (GM-CSF, Gibco) and 800 U/mL interleukin (IL)-4 (Gibco) for 6 days.

### Cytotoxicity assessment

Cytotoxicity was estimated using the lactate dehydrogenase (LDH) assay (Cytotox 96® Non-Radioactive Cytotoxicity Assay Kit, Promega, 96-well plate format). HMDM (10^6^ cells/ml) were exposed to the nanomaterials at 10, 30 and 100 μg/mL in 200 μL for 24 h. After exposure, 50 μl of the supernatants was transferred to a new 96-well plate. The cells were washed and lysed with 100 μL 0.1% Triton and Lysis buffer for 45 min at 37 °C. Then, 50 μL of the lysate was transferred to a new 96-well plate and 50 μL of reconstituted substrate was added to the supernatants and the cell lysates. After 20 min incubation, the 50 μL of stop solution was added to each well and the absorbance was recorded at 495 nm using a Tecan Infinite F200 plate reader (Männendorf, Switzerland). The toxicity was expressed as the percent of LDH release in supernatant (absorbance of supernatant) compared to maximum LDH release (absorbance of supernatant + cell lysate). Possible interference with the assay was assessed by incubating nanomaterials with reconstituted LDH substrate. No interference was detected.

### Cellular uptake

HMDM were exposed to SWCNTs or GO in RPMI-1640 medium supplemented with FBS for 24 h and then washed with PBS, trypsinated, and centrifuged at 2000 rpm for 3 min. Cells were then fixed in 2% glutaraldehyde in 0.1 M sodium cacodylate buffer containing 0.1 M sucrose and 3 mM CaCl_2_, pH 7.4, and stored in the refrigerator. Cells were washed in buffer and postfixed in 2% osmium tetroxide in 0.07 M sodium cacodylate buffer containing 1.5 mM CaCl_2_, pH 7.4, at 4° C for 2 h, dehydrated in ethanol followed by acetone, and embedded in LX-112 (Ladd, Burlington, VT). Sections were contrasted with uranyl acetate followed by lead citrate and were examined in a Tecnai 12 Spirit Bio TWIN TEM (FEI Company, Eindhoven, The Netherlands) at 100 kV. Digital images were taken using a Veleta camera (Olympus Soft Imaging Solutions, GmbH, Münster, Germany).

### cDNA microarray

HMDM were seeded in 24-well plates at a density of 10^6^ cells/well in a final volume of 1 mL and treated with the indicated concentrations of SWCNTs or GO. Cells were collected to RNAlater buffer (Ambion) and total RNA isolated with RNAqueous Small Scale Phenol-Free Total RNA Isolation Kit (Ambion). The quality of the total RNA was determined by Agilent Bionalyzer (Agilent, Santa Clara, CA). For microarray analysis 250 ng of total RNA was processed with GeneChip 39 IVT Express Kit (part no. 901229) and hybridized to GeneChip Human Genome U219 array plate (Affymetrix, Santa Clara, CA) with specific protocols using the GeneTitan Hybridization, Wash and Stain Kit for 39 IVT Array Plates (P/N 901530). Affymetrix GeneChip Command Console 3.1 was used to control the process and to summarize probe cell intensity data. Hybridization quality was checked with Affymetrix GeneChip Command Console and Expression ConsoleTM 1.1 s.

### Transcriptomics data analysis

Gene expression data was normalized using the RMA method^69^, and R/Bioconductor 3.0 and mapped to Ensembl gene identifiers^70,71^. Data was log2 transformed for analysis and visualization. Replicates (1–3 for each treatment) were summarized by taking the average of the log2 transformed fold change. The microarray data were submitted to the Gene Expression Omnibus database (GEO accession no. GSE83516). Gene expression data of all genes which had average fold change of greater than 0.75 log2 scale in a 24 h treatment of either SWCNT or GO was visualized with a heatmap using the Multi Experiment Viewer software, version 4.8.1, with genes ordered according to average fold change of treatment versus control in SWCNTs. Gene signature and pathway enrichment analysis was performed with Molecular Signature Database (MSigDB version 4.0) using canonical pathway descriptions and the Fisher’s exact test^72^. Only signatures which had a significance of q < 0.05 after multiple testing correction and at least three significant genes are reported. Ingenuity Pathway Analysis (IPA) (application version 220217, content version 16542223) (license obtained from Ingenuity Systems, Redwood City, CA) was performed in order to interpret the mRNA sequencing data^73^. Both canonical pathway enrichment analysis and upstream regulator analysis were performed using the IPA tool. The cut-off p < 0.01 and the overlap of at least three significantly affected genes was used. In addition, regulators were filtered by activation Z-score and all regulators were required to be expressed in the gene expression samples that were analyzed.

### Multiplex array for chemokine detection

HMDM (10^6^ cells/ml) were exposed to the nanomaterials at the indicated concentrations for 24 h. Cell supernatant were then collected, centrifuged at 15.000 g for 5 min to remove nanomaterials and cell debris and stored at −80 °C. Supernatants were never refrozen. Chemokines were quantified by using a Bio-Plex Pro Human Cytokine Assay (Bio-Rad) and Bio-Plex® system and software following the manufacturer’s instructions^74^. The cytokine standards were reconstituted in RPMI-1640 cell culture medium supplemented with 10% FBS. For NF-*κ*B inhibition studies, HMDM were pre-exposed to Bay 11-7082 (10 µM) for 1 h. After pre-incubation, the inhibitor was removed from the medium and cells were exposed to SWCNTs.

### Chemokine detection by ELISA

To complement the multiplex array experiments, selected chemokines were also determined by using a specific enzyme-linked immunosorbent assay (ELISA). To this end, HMDM (10^5^ cells/well) were exposed to 30 µg/mL of SWCNTs for 12 h. For some experiments, cells were first pretreated or not with cytochalasin D (10 μM) for 2 h and then exposed to SWCNT for 12 h in the presence or absence of cytochalasin D. To block TLR signaling, cells were pretreated with oxPAPC (30 or 60 µg/mL) prior to exposure to SWCNTs (30 µg/mL). Additionally, to block MyD88 signaling, cells were preincubated with Pepinh-MYD (25 µM) or Pepinh-Control (25 µM) prior to exposure to SWCNTs. Then, the supernatants were collected and stored at −80 °C for subsequent analysis. CCL5 expression in the supernatants was determined using a specific ELISA (Human RANTES ELISA, Life Technologies, Sweden) following the manufacturers’ instruction. In brief, the cell culture supernatants (controls and SWCNT-exposed samples) or CCL5 standards were added to the pre-coated ELISA wells provided in the kit and incubated for 1 h at RT. Then, the wells were washed and incubated first with the biotinylated antibody reagent solution and then with the streptavidin-HRP solution for 1 h, respectively. Finally, the chromogenic TMB substrate solution was added and the plates were kept in the dark for 30 min, followed by stopping the reaction. The absorbance was measured at 450 nm in a Tecan plate reader. A standard curve of CCL5 was prepared and the amount of CCL5 in cell culture supernatants was calculated from the standard curve.

### NFkappaB activation

NF-κB activity was determined by the NF-κB p65 (pS536) PhosphoTracer ELISA kit (Abcam, Sweden) according to the manufacturer’s instruction. Briefly, HMDM (10^6^ cells/ml) were exposed to 30 µg/ml of SWCNTs or LPS (0.1 µg/mL) or medium alone for 12 h, in presence or absence of Pepinh-MYD (25 µM) or Pepinh-Control (25 µM). Cells were then harvested and washed with PBS and the phosphorylated NF-κB p65 (pS536) protein was quantified based on a horseradish peroxidase-ADHP substrate reaction. The fluorescence was read at ex. 540 nm and em. 590 nm using a Tecan plate reader.

### TLR reporter cell lines

HEK 293 cells co-transfected with human TLR2 (HEK-Blue™ hTLR2) or TLR4 (HEK-Blue™ hTLR4 cells) and an NF-κB/AP-1-secreted embryonic alkaline phosphatase (SEAP) reporter gene were obtained from InvivoGen (Toulouse, France). Once TLR signaling is initiated, NF-κB and AP-1 is activated, which initiates the secretion of SEAP which can be detected in the cell supernatants to quantify NF-κB activation. The HEK-Blue™ Null1 cells were included as a negative control. HEK-Blue™ Null1, hTLR2 and hTLR4 cells were cultured in DMEM growth medium containing 4.5 g/L glucose and supplemented with 10% FBS, 50 U/mL penicillin, 50 mg/mL streptomycin, 100 mg/mL Normocin™, 2 mM L-glutamine and 1 × HEK-Blue™ selection antibiotics mixture, according to the manufacturers’ instruction. For Null1 cells, Zeocin™ was used instead of the selection antibiotics mixture. Cells (2 × 10^5^ cells/mL) were exposed for 12 h to 30 µg/mL of SWCNTs or LPS (0.1 µg/mL) as a positive control in HEK-Blue™ detection medium (InvivoGen). SEAP activity was measured at 630 nm using an Infinite F200 Tecan plate reader.

### CCR5 receptor expression

CCR5 cell surface expression was assessed by flow cytometry. Briefly, 10^6^ cells were resuspended in 100 µL of FACS solution (0.1% bovine serum albumin plus 0.1% sodium azide in phosphate-buffered saline) and incubated with antibody or isotype control at +4°C in the dark. FITC-conjugated rat monoclonal anti-CCR5 antibody [HEK/1/85] (Abcam, ab11466, lot GR60886-7) and rat IgG2a (BD Pharmingen) (isotype control) were used. After incubation, cells were washed three times with cold FACS solution and fixed with 2% formaldehyde for 20 min at RT. Cells were immediately analyzed using a FACScan flow cytometer (Becton Dickinson, San Jose, CA) operating with FBS Express 4 Flow Cytometry software; 10.000 events were collected for each sample.

### Cell migration assay

Cell migration was evaluated using a 24-transwell chemotaxis microchambers (Costar). To this end, 600 µL of medium (RPMI-1640 with 10% FBS) or conditioned medium (supernatant of 100 µg/mL SWCNT-exposed or 100 µg/ml GO-exposed HMDM) was added to the lower wells of a chemotaxis chamber. A polycarbonate 5 µm pore size filter was layered onto the wells, and 100 µL of cell suspension (10^5^ cells/mL of monocytes or DCs) were seeded in the upper chamber. The plate was covered and incubated at 37 °C for 3 h. At the end of the incubation, filters were removed, and the migrated cells were counted in the lower chamber. Results were expressed as the chemotactic index: the mean number of cells migrating to a test stimulus (SWCNT- or GO-conditioned medium) divided by the number of cells migrated to the control (medium alone).

### Molecular docking

The docking simulation of CNTs to TLRs was performed using the AutoDock Vina software^75^. The 3D structures of model nanotubes, i.e., zigzag nanotubes with chirality parameters (14,0) and a corresponding diameter of 1.1 nm were generated using the TubeGen 3.4 tool^76^. The length of each CNT was 8 nm. For oxidized CNTs, carboxylated groups were added on both ends (until saturation) and on the surface of the nanotube (randomly) using the Molfacture plugin in VMD visualization software^77^. The target structure was taken from the x-ray crystal structure of TLR4 (PDB code: 3fxi, chain A) and a 1 Å-spaced grid map with 200 × 200 × 200 points was built around the protein to search for possible binding sites over the entire surface of the target. Gasteiger partial atom charges in both protein and nanotube structures were assigned by using the AutoDock Tools suite program^78^. Docking simulations were carried out with a maximum number of 1000 generated binding modes. The top orientations were selected with a maximum energy difference of 10 kcal/mol between the best and worst retained binding modes.

### Statistics

All experiments were performed in at least three biological replicates and at least in technical duplicates; data are shown as average ± S.D. Unpaired two-tailed Student’s t-test or one-way ANOVA with post-hoc Tukey’s test was used for statistical analysis.

### Data availability

The data that support the findings of this study are reported in the article and in the supplementary information files and from the corresponding author upon request. The transcriptomics data were submitted to the Gene Expression Omnibus database.

## Electronic supplementary material

Supporting Information

Supplementary Tables 1-2

## References

[1] Bhattacharya K (2016). Biological interactions of carbon-based nanomaterials: from coronation to degradation. Nanomedicine.

[2] Boraschi, D., Fadeel, B. & Duschl, A. Immune System. In: *Adverse Effects of Engineered Nanomaterials: Exposure, Toxicology and Impact on Human Health (Second Edition)*. Eds. Fadeel, B., Pietroiusti, A., Shvedova, A. pp. 313–337. Elsevier (2017).

[3] Fadeel B (2012). Clear and present danger? Engineered nanoparticles and the immune system. Swiss Med Wkly.

[4] Deng ZJ, Liang M, Monteiro M, Toth I, Minchin RF (2011). Nanoparticle-induced unfolding of fibrinogen promotes Mac-1 receptor activation and inflammation. Nat Nanotechnol.

[5] Castranova V, Schulte PA, Zumwalde RD (2013). Occupational nanosafety considerations for carbon nanotubes and carbon nanofibers. Acc Chem Res.

[6] Shvedova AA (2005). Unusual inflammatory and fibrogenic pulmonary responses to single-walled carbon nanotubes in mice. Am J Physiol Lung Cell Mol Physiol.

[7] Tkach AV (2011). Direct effects of carbon nanotubes on dendritic cells induce immune suppression upon pulmonary exposure. ACS Nano.

[8] Grosse Y (2014). Carcinogenicity of fluoro-edenite, silicon carbide fibres and whiskers, and carbon nanotubes. Lancet Oncol.

[9] Kuempel ED (2017). Evaluating the mechanistic evidence and key data gaps in assessing the potential carcinogenicity of carbon nanotubes and nanofibers in humans. Crit Rev Toxicol.

[10] Bianco A, Prato M (2015). Safety concerns on graphene and 2D materials: a Flagship perspective. 2D Mater.

[11] Mukherjee SP, Bottini M, Fadeel B (2017). Graphene and the immune system: a romance of many dimensions. Front Immunol.

[12] Roberts JR (2016). Evaluation of pulmonary and systemic toxicity following lung exposure to graphite nanoplates: a member of the graphene-based nanomaterial family. Part Fibre Toxicol.

[13] Kim JK (2016). 28-Day inhalation toxicity of graphene nanoplatelets in Sprague-Dawley rats. Nanotoxicology.

[14] Ali-Boucetta H (2013). Purified graphene oxide dispersions lack *in vitro* cytotoxicity and *in vivo* pathogenicity. Adv Healthc Mater.

[15] Orecchioni M (2016). Molecular and genomic impact of large and small lateral dimension graphene oxide sheets on human immune cells from healthy donors. Adv Healthc Mater.

[16] Gay NJ, Symmons MF, Gangloff M, Bryant CE (2014). Assembly and localization of Toll-like receptor signalling complexes. Nat Rev Immunol.

[17] Li Y, Boraschi D (2016). Endotoxin contamination: a key element in the interpretation of nanosafety studies. Nanomedicine (Lond).

[18] Mukherjee SP (2016). Detection of endotoxin contamination of graphene based materials using the TNF-α expression test and guidelines for endotoxin-free graphene oxide production. PLoS One.

[19] Tuomela S (2013). Gene expression profiling of immune-competent human cells exposed to engineered zinc oxide or titanium dioxide nanoparticles. PLoS One.

[20] Zhang Y (2014). Modular analysis of bioinformatics demonstrates a critical role for NF-κB in macrophage activation. Inflammation.

[21] Kawai T, Akira S (2010). The role of pattern-recognition receptors in innate immunity: update on Toll-like receptors. Nat Immunol.

[22] Erridge C, Kennedy S, Spickett CM, Webb DJ (2008). Oxidized phospholipid inhibition of toll-like receptor (TLR) signaling is restricted to TLR2 and TLR4: roles for CD14, LPS-binding protein, and MD2 as targets for specificity of inhibition. J Biol Chem.

[23] Loiarro M (2005). Peptide-mediated interference of TIR domain dimerization in MyD88 inhibits interleukin-1-dependent activation of NF-*κ*B. J Biol Chem.

[24] Andón FT (2017). Hollow carbon spheres trigger inflammasome-dependent IL-1β secretion in macrophages. Carbon.

[25] Meller S (2015). T(H)17 cells promote microbial killing and innate immune sensing of DNA via interleukin 26. Nat Immunol.

[26] Love RJ, Jones KS (2013). The recognition of biomaterials: pattern recognition of medical polymers and their adsorbed biomolecules. J Biomed Mater Res A.

[27] Foti M (1999). Upon dendritic cell (DC) activation chemokines and chemokine receptor expression are rapidly regulated for recruitment and maintenance of DC at the inflammatory site. Int Immunol.

[28] Tuttle DL, Harrison JK, Anders C, Sleasman JW, Goodenow MM (1998). Expression of CCR5 increases during monocyte differentiation and directly mediates macrophage susceptibility to infection by human immunodeficiency virus type 1. J Virol.

[29] Park KH, Chhowalla M, Iqbal Z, Sesti F (2003). Single-walled carbon nanotubes are a new class of ion channel blockers. J Biol Chem..

[30] Calvaresi M, Furini S, Domene C, Bottoni A, Zerbetto F (2015). Blocking the passage: C_60_ geometrically clogs K^+^ channels. ACS Nano.

[31] Turabekova M (2014). Immunotoxicity of nanoparticles: a computational study suggests that CNTs and C60 fullerenes might be recognized as pathogens by Toll-like receptors. Nanoscale.

[32] Ma P-C (2010). Dispersion, interfacial interaction and re-agglomeration of functionalized carbon nanotubes in epoxy composites. Carbon.

[33] Shvedova AA, Kagan VE, Fadeel B (2010). Close encounters of the small kind: adverse effects of man-made materials interfacing with the nano-cosmos of biological systems. Annu Rev Pharmacol Toxicol.

[34] Fadeel B, Fornara A, Toprak MS, Bhattacharya K (2015). Keeping it real: the importance of material characterization in nanotoxicology. Biochem Biophys Res Commun.

[35] Westmeier, D., Knauer, S. K., Stauber, R. H. & Docter, D. Bio-Nano Interactions. In: *Adverse Effects of Engineered Nanomaterials: Exposure, Toxicology and Impact on Human Health (Second Edition)*. Eds. Fadeel, B., Pietroiusti, A. & Shvedova A. pp. 3–14. Elsevier (2017).

[36] Donaldson K, Poland CA (2013). Nanotoxicity: challenging the myth of nano-specific toxicity. Curr Opin Biotechnol.

[37] Gallud A, Fadeel B (2015). Keeping it small: towards a molecular definition of nanotoxicology. Eur. J. Nanomed.

[38] Miao Y (2014). Nanoparticle as signaling protein mimic: robust structural and functional modulation of CaMKII upon specific binding to fullerene C_60_ nanocrystals. ACS Nano.

[39] Kodali V (2013). Dysregulation of macrophage activation profiles by engineered nanoparticles. ACS Nano.

[40] Farrera C, Fadeel B (2015). It takes two to tango: understanding the interactions between engineered nanomaterials and the immune system. Eur J Pharm Biopharm.

[41] Ho CC (2013). Quantum dots induced monocyte chemotactic protein-1 expression via MyD88-dependent Toll-like receptor signaling pathways in macrophages. Toxicology.

[42] Shokouhi B (2010). The role of multiple toll-like receptor signalling cascades on interactions between biomedical polymers and dendritic cells. Biomaterials.

[43] Dumortier H (2013). When carbon nanotubes encounter the immune system: desirable and undesirable effects. Adv Drug Deliv Rev.

[44] Qu G (2013). Graphene oxide induces toll-like receptor 4 (TLR4)-dependent necrosis in macrophages. ACS Nano.

[45] Hari A (2014). Activation of NLRP3 inflammasome by crystalline structures via cell surface contact. Sci Rep.

[46] Ma J (2015). Crucial role of lateral size for graphene oxide in activating macrophages and stimulating pro-inflammatory responses in cells and animals. ACS Nano.

[47] McIntyre J (2016). A comparison of catabolic pathways induced in primary macrophages by pristine single walled carbon nanotubes and pristine graphene. RSC Adv.

[48] Fleischer CC, Payne CK (2014). Secondary structure of corona proteins determines the cell surface receptors used by nanoparticles. J Phys Chem B.

[49] Shannahan JH (2013). Comparison of nanotube-protein corona composition in cell culture media. Small.

[50] O’Connell DJ (2015). Characterization of the bionano interface and mapping extrinsic interactions of the corona of nanomaterials. Nanoscale.

[51] Saha K (2016). Regulation of macrophage recognition through the interplay of nanoparticle surface functionality and protein corona. ACS Nano.

[52] Simberg D (2009). Differential proteomics analysis of the surface heterogeneity of dextran iron oxide nanoparticles and the implications for their *in vivo* clearance. Biomaterials.

[53] Mu Q (2008). Protein binding by functionalized multiwalled carbon nanotubes is governed by the surface chemistry of both parties and the nanotube diameter. J. Phy. Chem C.

[54] Cai X (2013). Characterization of carbon nanotube protein corona by using quantitative proteomics. Nanomedicine.

[55] Zhao X (2015). Exploring the diameter and surface dependent conformational changes in carbon nanotube-protein corona and the related cytotoxicity. J Hazard Mater.

[56] Zlotnik A, Yoshie O (2000). Chemokines: a new classification system and their role in immunity. Immunity.

[57] Bhattacharya K, Andón FT, El-Sayed R, Fadeel B (2013). Mechanisms of carbon nanotube-induced toxicity: focus on pulmonary inflammation. Adv Drug Deliv Rev.

[58] Park EJ (2013). CCR5 plays an important role in resolving an inflammatory response to single-walled carbon nanotubes. J Appl Toxicol.

[59] Frank EA, Birch ME, Yadav JS (2015). MyD88 mediates *in vivo* effector functions of alveolar macrophages in acute lung inflammatory responses to carbon nanotube exposure. Toxicol Appl Pharmacol.

[60] Fujita K (2015). Intratracheal instillation of single-wall carbon nanotubes in the rat lung induces time-dependent changes in gene expression. Nanotoxicology.

[61] Kinaret P (2017). Inhalation and oropharyngeal aspiration exposure to rod-like carbon nanotubes induce similar airway inflammation and biological responses in mouse lungs. ACS Nano.

[62] Chen S (2016). No involvement of alveolar macrophages in the initiation of carbon nanoparticle induced acute lung inflammation in mice. Part Fibre Toxicol.

[63] Katwa P (2012). A carbon nanotube toxicity paradigm driven by mast cells and the IL-33/ST_2_ axis. Small.

[64] Shi J (2012). Microsomal glutathione transferase 1 protects against toxicity induced by silica nanoparticles but not by zinc oxide nanoparticles. ACS Nano.

[65] Gorelik, O., Nikolaev, P. & Arepalli, S. *Purification procedures for single-walled carbon nanotubes. NASA contractor report. NASA/CR-2000–208–926;* Document ID 20040200957, NASA Technical Reports Server (NTRS) 2000 [http://ntrs.nasa.gov].

[66] Marcano DC (2010). Improved synthesis of graphene oxide. ACS Nano.

[67] Mukherjee, S.P., Kostarelos, K. & Fadeel, B. Cytokine profiling of primary human macrophages exposed to endotoxin-free graphene oxide: size-independent NLRP3 inflammasome activation. *Adv Healthc Mater* 2017 Dec 21. 10.1002/adhm.201700815. [Epub ahead of print]. 10.1002/adhm.201700815 29266859

[68] Feliu N (2012). Stability and biocompatibility of a library of polyester dendrimers in comparison to polyamidoamine dendrimers. Biomaterials.

[69] Irizarry RA (2003). Exploration, normalization, and summaries of high density oligonucleotide array probe level data. Biostatistics.

[70] Dai M (2005). Evolving gene/transcript definitions significantly alter the interpretation of GeneChip data. Nucleic Acids Res.

[71] Huber W (2015). Orchestrating high-throughput genomic analysis with Bioconductor. Nat Methods.

[72] Wang J, Duncan D, Shi Z, Zhang B (2013). WEB-based GEne SeT AnaLysis Toolkit (WebGestalt): update 2013. Nucleic Acids Res.

[73] Krämer A, Green J, Pollard J, Tugendreich S (2014). Causal analysis approaches in Ingenuity Pathway Analysis. Bioinformatics.

[74] Gallud A (2017). Macrophage activation status determines the internalization of mesoporous silica particles of different sizes: exploring the role of different pattern recognition receptors. Biomaterials.

[75] Trott O, Olson AJ (2009). AutoDock Vina: Improving the speed and accuracy of docking with a new scoring function, efficient optimization, and multithreading. J Comput Chem.

[76] Frey, J.T. & Doren, D.J. TubeGen 3.4. *University of Delaware, Newark DE*. Available at: http://turin.nss.udel.edu/research/tubegenonline.html (2011).

[77] Humphrey W, Dalke A, Schulten K (1996). VMD–Visual Molecular Dynamics. J Mol Graph.

[78] Morris G (2009). AutoDock4 and AutoDockTools4: automated docking with selective receptor flexibility. J Comput Chem.

