# Supplementary material for: Macrophage sensing of single-walled carbon nanotubes via Toll-like receptors
Source: Sci Rep. 2018 Jan 18;8:1115. doi: 10.1038/s41598-018-19521-9 (PMC5773626; doi:10.1038/s41598-018-19521-9)
Supplement: Supplementary file 1 — Supporting Information [file 41598_2018_19521_MOESM1_ESM.pdf]

Supporting Information

Macrophage sensing of single-walled carbon nanotubes *via* Toll-like receptors

Sourav P. Mukherjee, Olesja Bondarenko, Pekka Kohonen, Fernando T. Andón, Táňa Brzicová, Isabel Gessner, Sanjay Mathur, Massimo Bottini, Paolo Calligari, Lorenzo Stella, Elena Kisin, Anna Shvedova, Reija Autio, Heli Salminen-Mankonen, Riitta Lahesmaa & Bengt Fadeel[1,†]

[1]Nanosafety & Nanomedicine Laboratory, Division of Molecular Toxicology, Institute of Environmental Medicine, Karolinska Institutet, 17177 Stockholm, Sweden;

[†]Correspondence and requests for materials. E-mail: Bengt.Fadeel@ki.se

Running title: Carbon nanotubes trigger activation of Toll-like receptors.

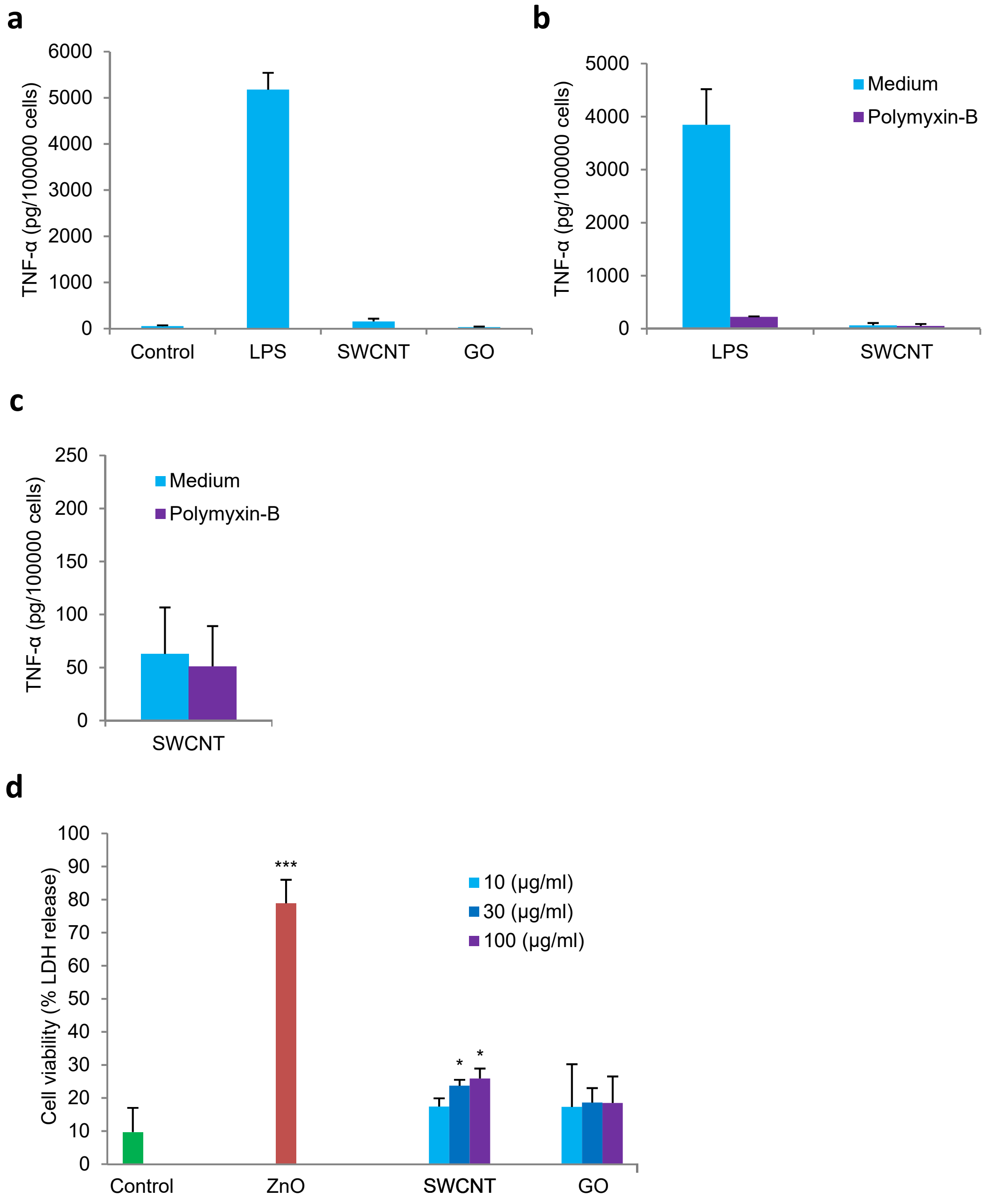

Figure S1. Endotoxin content and cytotoxicity assessment. a) Endotoxin content was assessed by using the TNF-α expression test (TET). HMDM were exposed for 24 h to SWCNTs or GO, or to LPS, and TNF-α secretion was measured by ELISA. b) TNF-α secretion measured in the presence or absence of the LPS inhibitor, polymyxin B (10 μM). c) The same results as in b) plotted without the positive control (LPS) (note: different scale). d) HMDM were exposed for 24 h to SWCNT or GO at the indicated concentrations, or to 100 μg/ml ZnO NPs (positive control) and cell viability was determined by using the LDH assay. Data are shown as mean values ± S.D. of three independent experiments using cells from different donors; p-values by Student's *t*-test, *<0.05; ***<0.001.

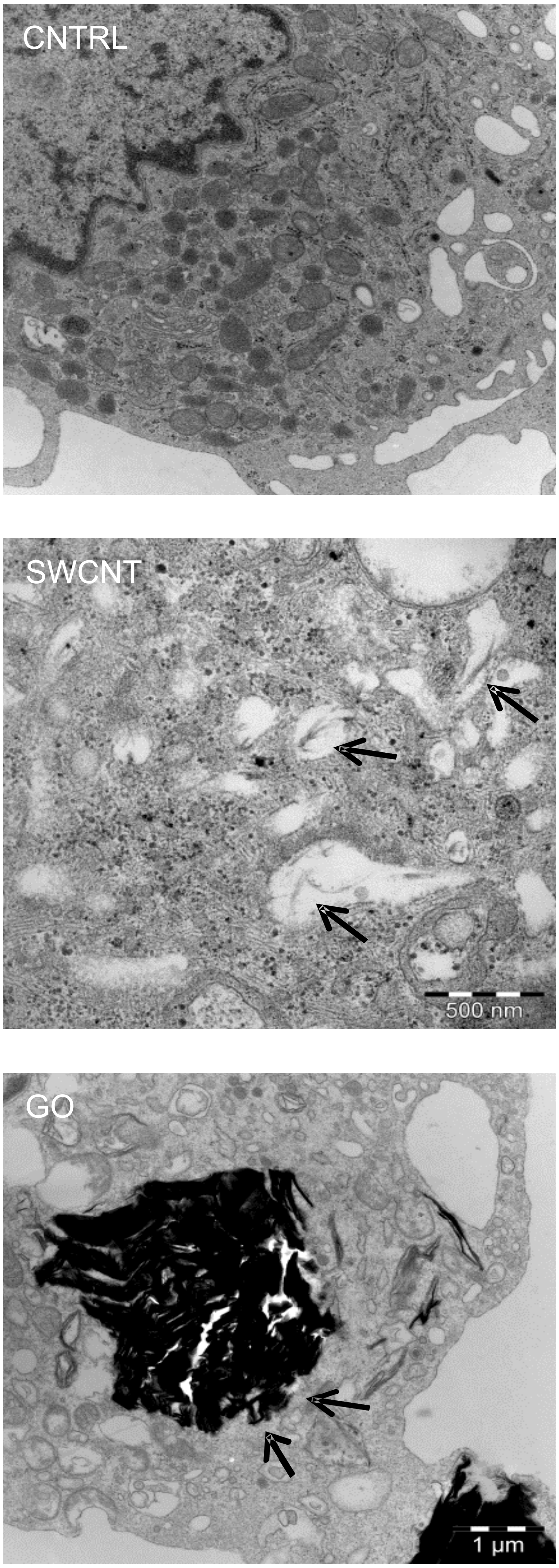

Figure S2. Macrophage uptake of SWCNT and GO. TEM micrographs of HMDM after 24 h exposure to medium alone, 100 µg/mL SWCNTs and 100 µg/mL GO. The arrows indicate the presence of nanomaterials in vesicular, membrane-enclosed structures.

a

b

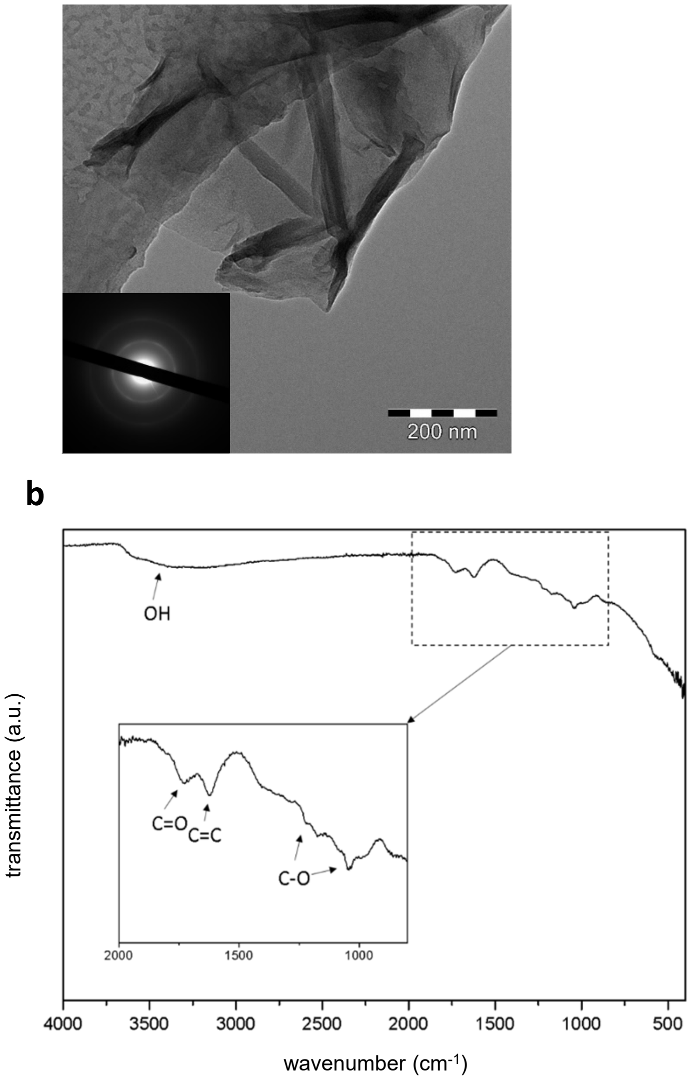

Figure S3. Characterization of graphene oxide. a) TEM demonstrated a flake-like morphology and thus a two-dimensional structure of the sample. Crystallinity of the sample was further demonstrated by electron diffraction pattern (inset). b) FT-IR spectrometric analysis revealed a broad band between 3600-3000 cm^{-1} which can be attributed to O-H stretching vibrations, typical for hydroxyl functions as well as for adsorbed water molecules on the surface. This broad band overlaps with the weak C-H stretching vibration around 2900 cm^{-1} while the strong band at 1618 cm^{-1} can be attributed to sp^2 hybridized carbon in the form of C=C bonds. Furthermore, a variety of characteristic C-O bands can be observed at 1726 cm^{-1} (C=O) and 1221 cm^{-1} as well as 1043 cm^{-1} (both C-O valence vibrations). c) XPS measurements revealed a carbon to oxygen ratio of roughly 2:1. Similar to FT-IR analyses, high resolution C1s measurements revealed the presence of C-C and C-H bonds as well as several functional C-O groups including aldehydes, ketones, carboxylic acids and esters (d). This was further supported by high resolution O1s measurements (e) whereby measured signals could be attributed to C=O double bonds, but also aliphatic and phenolic C-O single bonds as well as hydroxyl groups.

c

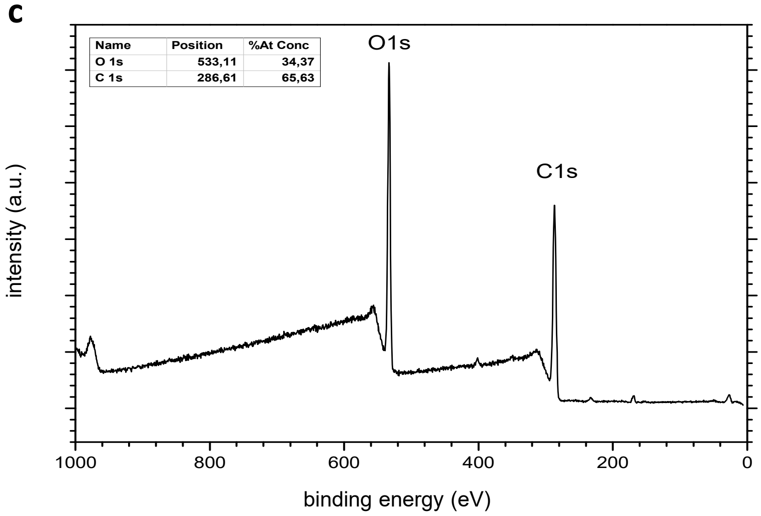

d

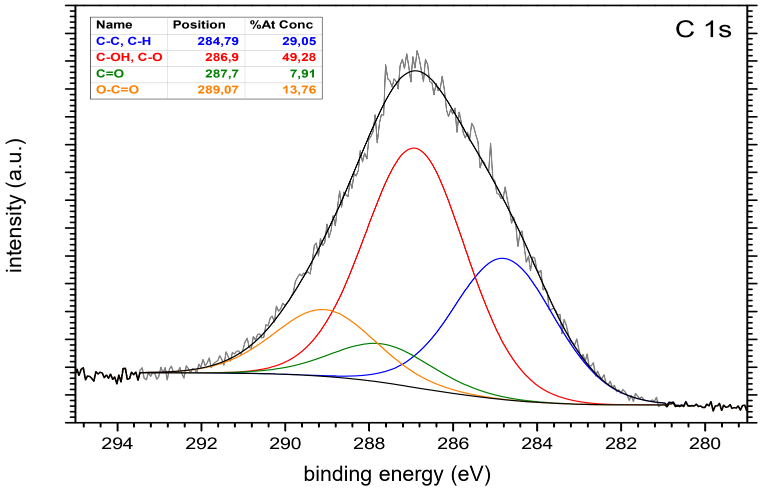

e

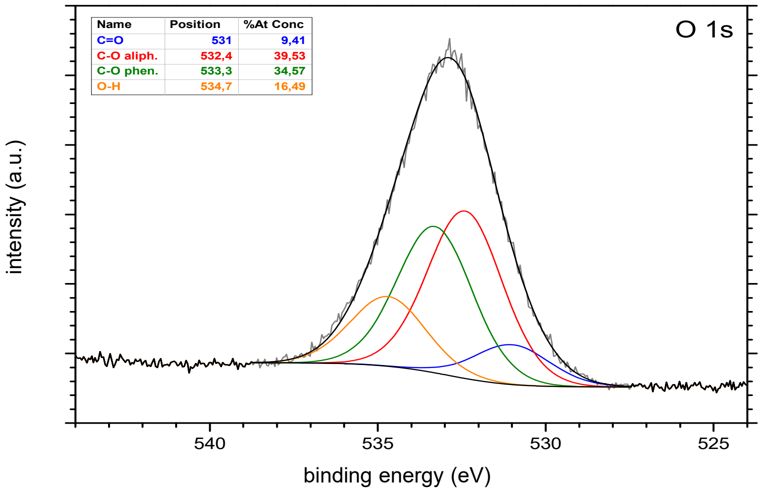

Figure S3

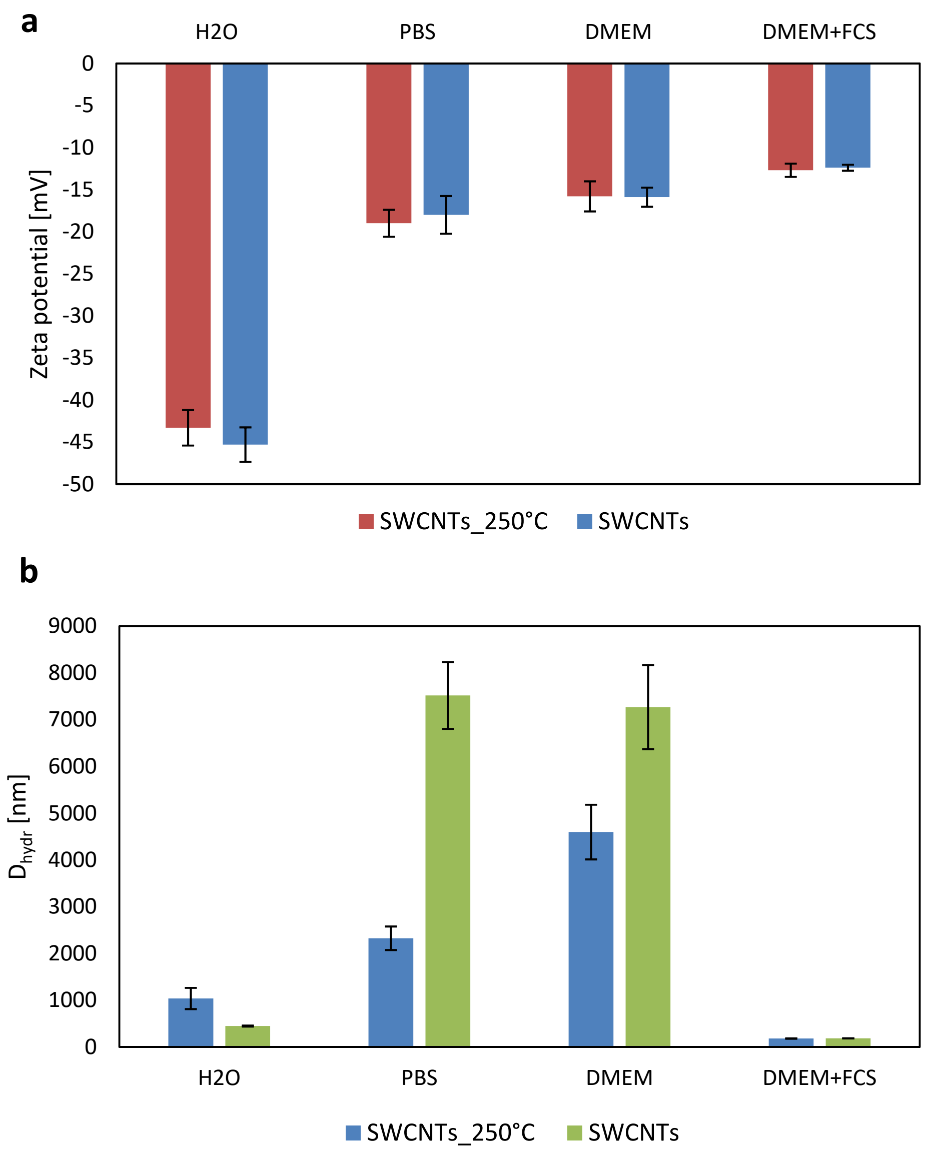

Figure S4. Characterization before/after calcination. SWCNTs were subjected to calcination at 250°C to remove endotoxin and samples were analyzed before and after calcination following dispersion in water, PBS, or cell culture medium (DMEM) with or without 10% FBS. a) ζ-potential and b) dynamic light scattering measurements.

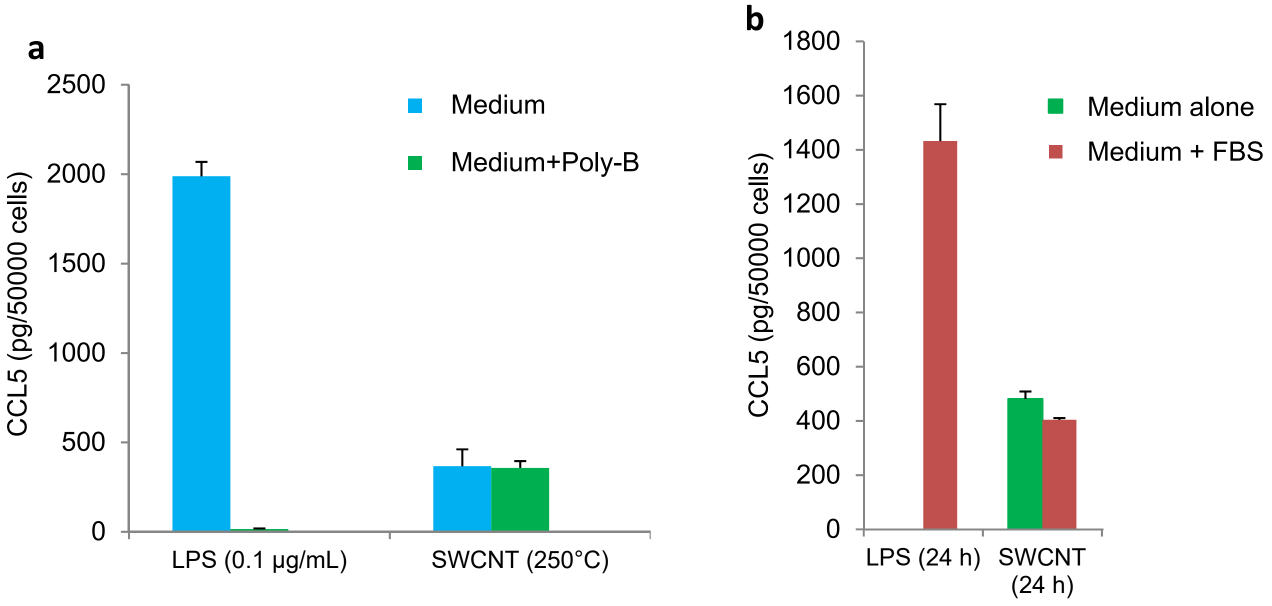

Figure S5. Role of calcination and serum. a) HMDM were exposed for 12 h to SWCNT (30 µg/mL) that had been subjected to calcination at 250°C, with or without co-incubation with the endotoxin inhibitor, polymyxin-B (10 µM), and CCL5 secretion was determined by ELISA. LPS was added as a positive control. b) HMDM were exposed for 24 h to SWCNTs in the presence or absence of 10% fetal bovine serum (FBS), or to LPS (0.1 µg/mL) as a positive control, and CCL5 secretion was measured by ELISA.

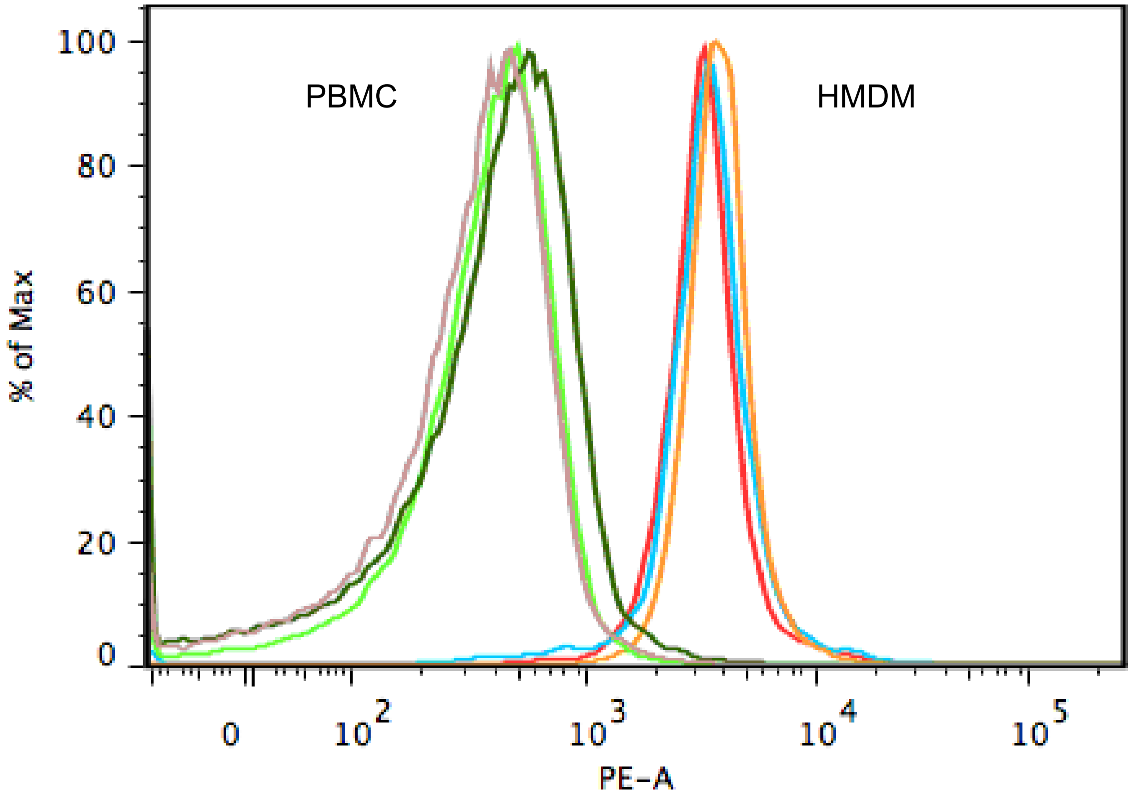

Figure S6. Macrophage differentiation. Freshly isolated peripheral blood mononuclear cells (PBMC) were cultured in RPMI-1640 medium supplemented with 10% FBS and penicillin-streptomycin in the presence of M-CSF for 3 days to produce human monocyte-derived macrophages (HMDM). Cells were stained using PE-conjugated CD11b antibody. Three replicates for each condition (PBMC and HMDM) are shown.
